# Applications of nanomaterials with enzyme-like activity for the detection of phytochemicals and hazardous substances in plant samples

**DOI:** 10.1186/s13020-024-01014-9

**Published:** 2024-10-08

**Authors:** Lei Xu, Mao-Ling Luo, Jing-Jing Dai, Huan Zhu, Peng Li, Dan Wang, Feng-Qing Yang

**Affiliations:** 1 School of Chemistry and Chemical Engineering, Chongqing University, Chongqing, 401331 People’s Republic of China; 2 State Key Laboratory of Quality Research in Chinese Medicine, Macau Centre for Research and Development in Chinese Medicine, Institute of Chinese Medical Sciences, University of Macau, Macau, China

**Keywords:** Nanozymes, Plant samples, Phytochemicals, Hazardous substances

## Abstract

Plants such as herbs, vegetables, fruits, and cereals are closely related to human life. Developing effective testing methods to ensure their safety and quantify their active components are of significant importance. Recently, nanomaterials with enzyme-like activity (known as nanozymes) have been widely developed in various assays, including colorimetric, fluorescence, chemiluminescence, and electrochemical analysis. This review presents the latest advances in analyzing phytochemicals and hazardous substances in plant samples based on nanozymes, including some active ingredients, organophosphorus pesticides, heavy metal ions, and mycotoxins. Additionally, the current shortcomings and challenges of the actual sample analysis were discussed.

## Background

Plants, such as Chinese herbal medicines (CHMs) and edible plants, play crucial roles in human life [1]. The bioactive components in plants, namely phytochemicals, are important for human health and disease prevention. They usually have antioxidant capacity that protects cells from oxidative stress, as well as anti-inflammatory, antibacterial, and anti-tumor activities to prevent diseases like cancer, inflammatory bowel disease, and metabolic syndrome [2–4]. To date, various analytical techniques have already been used to analyze phytochemicals, including high-performance liquid chromatography (HPLC), mass spectrometry (MS), capillary electrophoresis (CE), gas chromatography (GC) [5, 6], etc. Although these methods are accurate and sensitive, sample handling is sophisticated with long analysis time and high cost. Thus, the development of novel, rapid, and selective approaches for the analysis of phytochemicals in plants and their extracts is of great importance for quick and on-site detection.

Furthermore, plants are frequently exposed to a variety of chemicals that are harmful to the human body, impacting their usability. For instance, inappropriate discharge of industrial wastewater leads to the accumulation of heavy metal ions in the soil, which will inevitably be absorbed by plants during planting. Excessive intake of heavy metal ions is prone to cause neurological disorders, kidney and liver damage, cardiovascular disease, and cancer [7, 8]. Therefore, the Chinese Pharmacopoeia (2020 edition) has set limits for heavy metals in Chinese medicinal materials and tablets of plant species: arsenic (2 mg/kg), cadmium (1 mg/kg), copper (20 mg/kg) lead (5 mg/kg), and mercury (0.2 mg/kg) [9]. In addition, organophosphorus pesticides (OPs) are widely used to protect plants from pests during cultivation, giving rise to the presence of excessive pesticide residues [10]. Once entering the human body, they irreversibly inhibit cholinesterase activity, posing a hazard to the cardiovascular, nervous, and respiratory systems [11]. In response, the Ministry of Agriculture and Rural Affairs of China has established the maximum residue limits (MRLs) of OPs in food. For example, apples’ MRLs of dichlorvos, glyphosate, and chlorpyrifos are 0.1, 0.5, and 1 mg/kg, respectively (GB 2763–2021) [12]. Furthermore, it should also be noted that plants may become contaminated by certain toxins during storage, such as mycotoxins. These toxins are highly carcinogenic and difficult to be completely removed during processing because of their thermal stability [13]. The current analytical methods for detecting heavy metal ions, OPs, and mycotoxins, including HPLC, GC–MS, atomic absorption spectrometry (AAS), inductively coupled plasma mass spectrometry (ICP-MS), and ultra-performance liquid chromatography-tandem mass spectrometry (UPLC-MS/MS), require complex pretreatment and specialized operations [8, 10, 14]. Therefore, there is a necessity to develop simple, efficient, and sensitive methods to detect these hazardous substances in plants for on-site and rapid inspection.

**Fig. 1:**
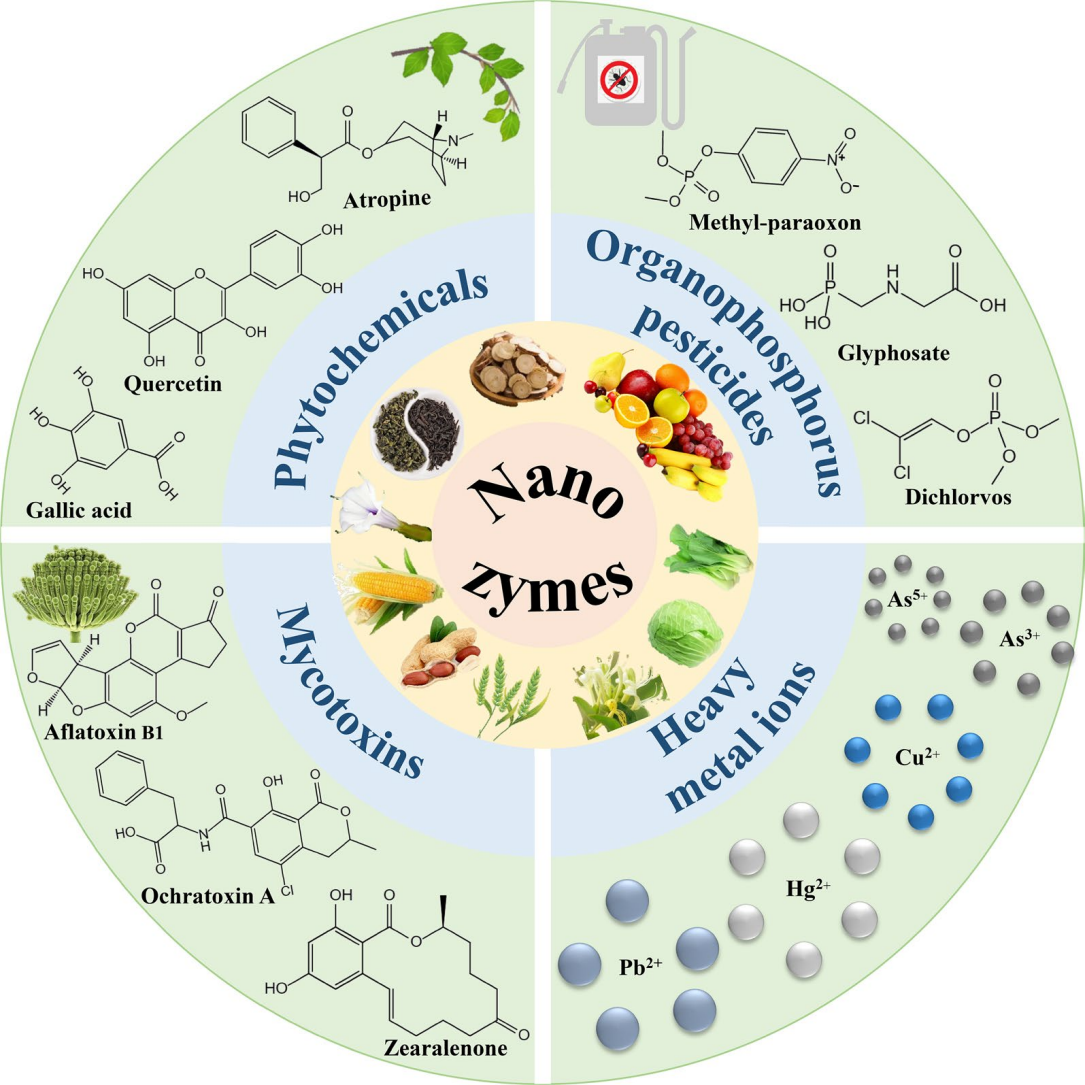
Review of nanozymes-based detection of phytochemicals and hazardous substances in plants

Nanozymes are a type of nanomaterials that exhibit enzyme-like activities, including metals (Au, Ag, Pt, Pd, etc.), metal oxides (CeO_2_, Fe_3_O_4_, Mn_3_O_4_, CuO, etc.), carbon-based compounds (carbon nanotubes, graphitic carbon nitride, carbon dots, etc.), and other nanomaterials (e.g., metal–organic frameworks (MOFs), covalent organic frameworks (COFs), metal sulfides, etc.) [15–17]. The enzyme-like activity of currently reported nanozymes can be mainly divided into two categories, oxidoreductase such as peroxidase (POD), oxidase (OXD), laccase (LAC), and superoxide dismutase (SOD), and hydrolase such as nuclease, esterase, phosphatase, and protease. Table 1 summarizes the functions of commonly reported nanozymes. Nanozymes have been widely used in the field of biomedicine, environmental monitoring, and food safety for their remarkable advantages of high stability, low cost, controllable activity, and easy storage [18, 19]. Most significantly, the nanozyme-based sensor offers the advantage of a shorter detection time that can fulfill the requirements of real-time detection [20–22]. For instance, Wang et al. [23] developed a manganese-based nanozyme that enabled rapid quantitative analysis of glutathione within 1 min. Xu et al. [24] synthesized copper-cobalt bimetallic nanozymes and combined with a smartphone and hydrogel kit to achieve real-time monitoring of perfluorooctane sulfonate (PFOS) in lake water. The approach offers a simpler instrument and quicker build-up compared to traditional methods like HPLC. Herein, this review aims to summarize recent advancements in applying nanomaterials with enzyme-like activity to detect phytochemicals and hazardous substances in plants (Fig. 1). Firstly, the application of nanozymes for detecting active phytochemicals was introduced, including gallic acid, tannic acid, ascorbic acid, rutin, atropine, quercetin, astragaloside-IV, and licorice. Secondly, advancements in the utilization of nanozymes for detecting hazardous substances in plants were presented, such as organophosphorus pesticides, heavy metal ions, and mycotoxins. Finally, the challenges and prospects in nanozyme-based detection of plant samples were discussed. This paper may provide useful information for readers to understand the design, performance, and application of nanozymes, to develop efficient, rapid, highly sensitive, and selective methods for detecting target components in actual plant samples.

**Table 1:** Summary of functions of currently reported nanozymes

Activity	Catalytic function	Refs.
POD	Catalyzing H_2_O_2_ to produce reactive oxygen species, and, subsequently, oxidizing the substrate (e.g., TMB)	[29, 32, 35, 36, 42, 51]
OXD	Activating O_2_ to yield reactive oxygen species, and then oxidizing the substrate (e.g., TMB)	[28, 37, 38, 47]
LAC	Oxidizing polyphenols and polyamines	[39, 46, 145]
SOD	Catalyzing the disproportionation of superoxide anion radical (O_2_^−^) to H_2_O_2_ and O_2_	[143]
Phosphatase	Hydrolyzing phosphate monoesters to remove the phosphate group from the substrate molecule, and generating phosphate ions and free hydroxyl groups	[124, 125, 128, 129]

POD, peroxidase; OXD, oxidase; LAC, laccase; SOD, superoxide dismutase; TMB, 3, 3’, 5, 5’-tetramethylbenzidine

## Detection of phytochemicals

Phytochemicals are biologically active secondary metabolites produced by plants for self-protection, including carotenoids, polyphenols, alkaloids, saponins, and others [25], which are obtainable from a variety of sources, such as herbs, vegetables, fruits, and teas [26]. Most of them exhibit potent antioxidant activity and contribute to reducing the risks of heart disease, cancer, diabetes, and other diseases [25, 27]. However, plants are intricate systems containing a multitude of substances, making it challenging to achieve specific analysis and identification of the target phytochemicals. Table 2 summarizes some of the studies on the detection of phytochemicals in plants by nanozyme-based methods.

**Table 2:** Summary of detection of phytochemicals in plants based on nanozymes

Nanozyme/activity	Analyte	Method	Sample	LOD(µM)	Linear range(µM)	Refs.
Cu-Guo NRs/LAC	Rutin	Colorimetric	Propolis, Rutin-containing dietary supplement tablets, urine, and blood serum	0.114	0.77–54.46	[46]
CTF–1/OXD	Rutin	Chemiluminescence	Tablets and Flos Sophorae Immaturus	0.015	0.03–0.25	[47]
Fe_3_O_4_@MOF/Dextrin/POD	Atropine	Fluorescence	*Datura stramonium* and *D. innoxia*	2.27 μg/L	1–600 μg/L	[40]
Fe_3_O_4_@Zn/Mg MOF/POD	Atropine	Chemiluminescence	*Datura stramonium* and *D. innoxia*	0.02 μg/L	5–600 μg/L	[48]
Iron oxide/POD	Glycyrrhizic acid/liquiritin/licochalcone A/isolicoflavonol	Colorimetric sensor array	*Glycyrrhiza uralensis*	–	1–200	[56]
Mb(Cu^II^)-AuNPs/POD and PPO	Gallic acid	Electrochemistry	Black tea, grapes, and oranges	0.27	1–1000	[34]
LaFeO_3_/POD	Gallic acid	Colorimetric	Green tea, diet tea, and pharyngitis tablets	0.4	0.67–40.8	[29]
N-Mn_3_O_4_ NSps/OXD	Gallic acid	Colorimetric	Black tea and green tea	0.028	5–30	[28]
VB_6_/POD	Gallic acid/H_2_O_2_	Colorimetric	Oolong tea, black tea, and green tea/Milk	4.1/12.1	10–50/50–600	[146]
CoOOH nanorings/OXD	Gallic acid	Colorimetric	Green tea	0.025	0.25–20	[37]
CeO_2_/Co_3_O_4_@NCH/POD	Quercetin/H_2_O_2_	Colorimetric	Yinxingye Dispersible Tablets	1.19/86	7–22/400–1000	[42]
Cu-TA NSs/LAC	Quercetin	Colorimetric	Green pepper, dill, and red onion	0.064	0.35–32.09	[39]
Ar-MoO_3_NPs/POD	Quercetin/resveratrol/curcumin/gallic acid/ellagic acid	Fluorescence	Apple, orange, and grape	12.22/61.89/38.89/21.5/16.25	2–232/2–270/39–400/2–309/39–309	[44]
AuNCs-p-h/POD	Tea polyphenols	Colorimetric	Huangshan Maofeng, Tongqin green tea, Sanxia Jianhao, and Lipton tea	0.01	0.01–10	[147]
Cu/CN/POD	Tannic acid	Colorimetric	Green tea and Pu’er tea	0.03	0.09–3.2	[30]
SrTiO_3_-rGO/POD	Tannic acid	Colorimetric	Green tea and Oolong tea	0.056	1–100	[32]
Fe-HHTP/POD	Tannic acid	Colorimetric	Teas (Green tea and Pu’er tea) and red wines (La suerte and Great wall)	0.5	0.5–100	[31]
CuS HNCs/POD	Tannic acid	Colorimetric/ photothermal/RGB	Green tea, red tea, and Oolong tea	0.08/0.13/0.25	1–20/1–10/1–10	[35]
MnO_2_/GQD/OXD	Gallic acid/tannic acid/ascorbic acid	Colorimetric	Mango juice, lemon juice, and black tea	0.07/0.28/0.69	5–25/1–5/6–80	[38]
Pd-Pt-Ru/POD	Ascorbic acid/H_2_O_2_	Colorimetric	Drinks, foods, and herbs (*Cornus officinalis*, *Cynanchum otophyllum*, *Dioscorea bulbifera*, and Eriobotryae Folium)	1.13/2790	2–12/5000–4 × 10^4^	[36]
MIP@PDA/CuO NPs/POD	Astragaloside-IV	Colorimetric	Huangqi Granules and Ganweikang Tablets	0.000991 mg/mL	0.000341–1.024 mg/mL	[51]

POD, peroxidase; OXD, oxidase; LAC, laccase; PPO, polyphenol oxidase; NPs, nanoparticles; NSs, nanosheets; AuNPs, gold nanoparticles; N-Mn_3_O_4_ NSps, nitrogen-doped Mn_3_O_4_ nanospheres; Ar-MoO_3_NPs, molybdenum trioxide nanoparticles by Argon cold plasma surface modification; Cu-Guo NRs, Cu-guanosine nanorods; Cu/CN, carbon nitride-supported Cu single-atom nanozymes; rGO, reduced graphene oxide; Fe-HHTP, Fe-2, 3, 6, 7, 10, 11-Hexahydroxytriphenylene; HNCs, hollow nanocages; GQD, graphene quantum dot; AuNCs-p-h, protein conjugated gold nanoclusters under heating conditions; NCH, N-doped hollow carbon microspheres; Cu-TA, Cu-tannic acid; Mb, methanobactin; CTF-1, covalent triazine framework; MOF, metal–organic framework; MIP, molecularly imprinted polymer; PDA, polymerized dopamine; VB_6_, vitamin B6

### Direct detection

Gallic acid (GA) and tannic acid (TA) are a class of natural phenolic compounds widely found in fruits and teas with various biological activities, such as antioxidant, anticancer, anti-mutagenesis, and antiviral [28–31]. The nanomaterials with POD-like and OXD-like activities can catalyze the oxidation of the substrate 3, 3’, 5, 5’-tetramethylbenzidine (TMB) to generate blue oxidized TMB (ox-TMB) in the presence of H_2_O_2_ and O_2_, respectively. GA and TA can inhibit the oxidation of TMB due to their antioxidant property, realizing colorimetric detection of them. Besides, instead of complicated pretreatment, these active ingredients can be directly detected in the real samples through a simple water extraction. Perovskite is a type of transition metal oxide, some of which possess splendid catalytic activity. Chen et al. [29] developed a simple colorimetric method to detect GA based on the POD-like activity of LaFeO_3_ microspheres, which is a typical perovskite, with a linear range of 0.67–40.8 µM and a limit of detection (LOD) of 0.4 µM. In addition, the established method was used in the determination of GA in diet tea, green tea, and pharyngitis tablets with good recoveries of 95.65–102.10% and RSD (*n* = 3) less than 4.00%. The activity of nanozymes plays a vital role in detecting phytochemicals, which can affect the detection sensitivity. Combining carbon-based materials with perovskite can enhance their catalytic performance. Liu et al. [32] synthesized the heterojunctions composed of strontium titanate (SrTiO_3_) and reduced graphene oxide (rGO), which facilitate photo-generated charge transfer under ultraviolet irradiation, resulting in an excellent POD-like activity. It is noted that the affinity for TMB of SrTiO_3_-rGO composites is 19 times higher than that of natural horseradish peroxidase (HRP). Meanwhile, the colorimetric quantitative detection of TA shows a lower LOD of 0.056 µM, which has been successfully applied to detect TA in green tea and Oolong tea.

**Fig. 2:**
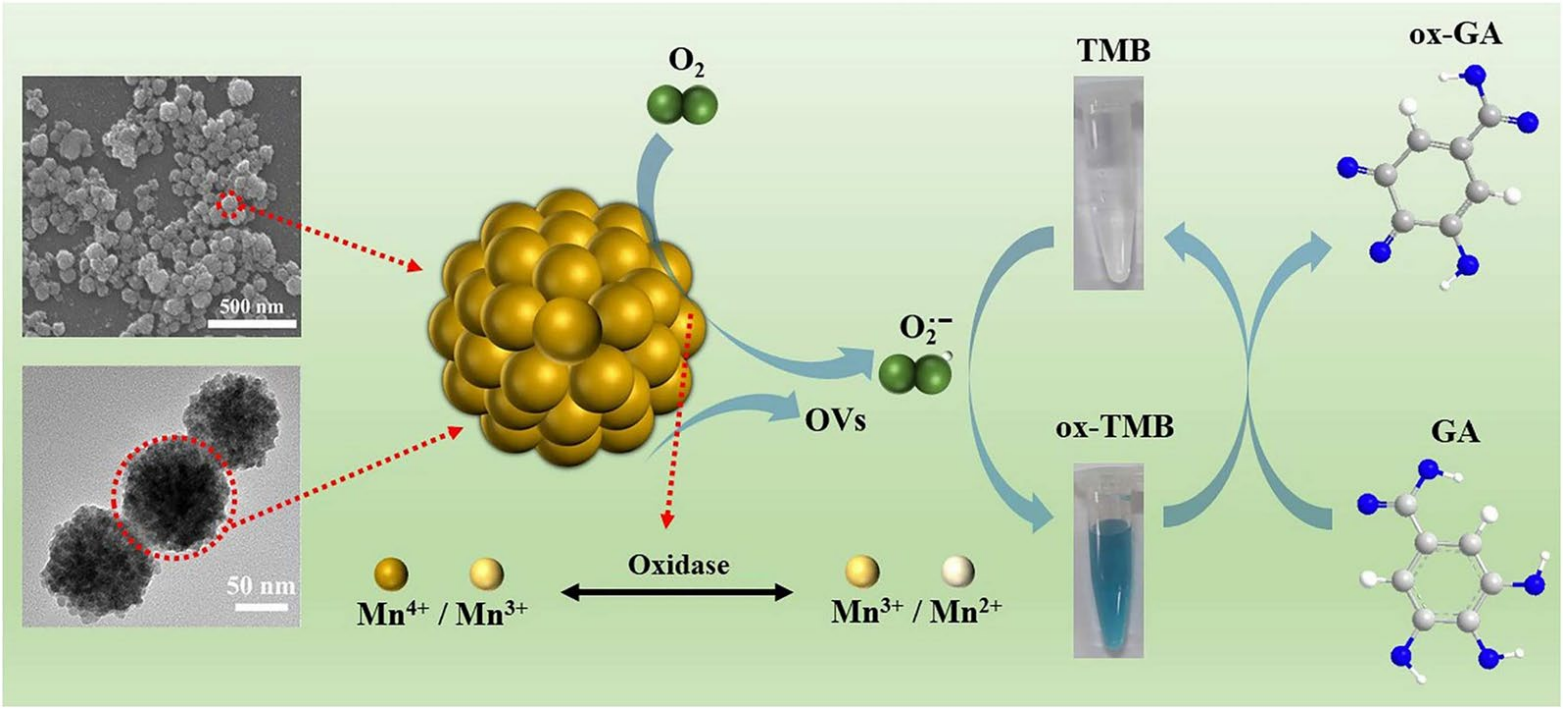
Schematic diagram of detecting GA based on N-Mn_3_O_4_ NSs. Reprinted with permission from [28]

Atomic doping is also one of the methods to enhance enzyme-like activity. Furthermore, oxygen vacancies (OVs) are a kind of metal oxide defects, which are formed by the detachment of oxygen from the lattice of metal oxides in a specific external environment (e.g., high temperature). OVs can provide rich active sites and high surface energy to improve the catalytic activity of nanozymes [33]. Zhou et al. [28] prepared a raspberry-like nitrogen-doped Mn_3_O_4_ nanospheres (N-Mn_3_O_4_ NSps) with OVs, which exhibited enhanced OXD-like activity (Fig. 2). The senor based on N-Mn_3_O_4_ NSps showed excellent reproducibility, stability, and interference resistance for detecting GA with a linear range of 5–30 µM and a lower LOD of 0.028 µM, which is feasible for the detection of GA both in green tea and black tea with the RSD (*n* = 5) within 3.27%. Furthermore, a platform based on the smartphone was implemented for GA detection with a LOD of 0.047 µM.

In addition, the electrochemical assay has also been exploited for the quantification of GA. Based on gold nanoparticles, Chen et al. [34] developed a peptide-modified dual mimetic enzyme sensor for the detection of GA. The construction mechanism relied on the active center of the methanobactin (Mb) structure that can capture Cu(II), resulting in the coordinated complex Mb(Cu^II^) with polyphenol oxidase (PPO)- and POD-like activities. After the addition of GA, the sensor with surface-modified gold nanoparticles and Mb(Cu^II^) exhibited a high oxidation peak with a peak potential of 0.79 ± 0.05 V. Subsequently, the developed method was employed to detect GA in three real samples, including grapes, oranges, and black tea, with recoveries of 96.76–100.95% and RSD (*n* = 3) less than 5%.

**Fig. 3:**
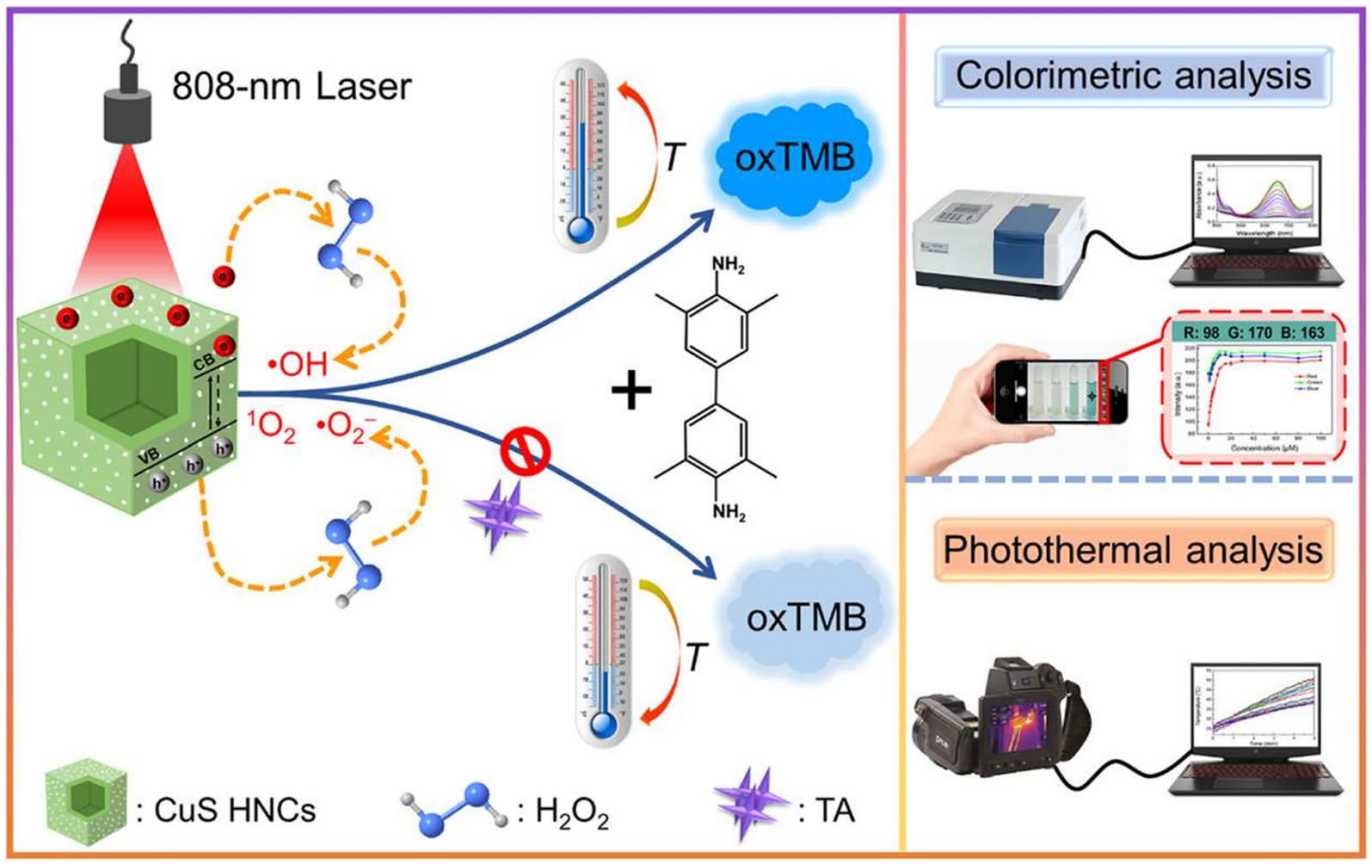
Schematic mechanism for dual-mode detection of TA based on the CuS HNC probe. Reprinted with permission from [35]

To explore additional response mechanisms is an effective approach to improve the detection selectivity. The 2,3,6,7,10,11-Hexahydroxytriphenylene (HHTP) is a highly conjugated triol ester that can coordinate with a metal-based node to form two-dimensional porous expansion frameworks known as metal catecholates (M-CATs). Inspired by the structure of M-CATs, Wu et al. [31] prepared a Fe-HHTP amorphous nanomaterial with POD-like activity through an one-step self-assembly strategy. The colorimetric method based on Fe-HHTP can rapidly detect TA within the linear range of 0.5–100 µM with a LOD of 0.5 µM, which was successfully used to measure TA content in tea and red wine samples. Remarkably, the inhibition of TA on the color reaction was resulted not only from its antioxidant ability but also from the formation of a Fe^3+^-TA complex. However, GA and AA still exhibited certain interference on the detection of TA. To further address this issue, Wu et al. [35] developed a colorimetric/photothermal dual-mode analysis method for TA detection based on the light-enhanced POD-like activity and high photothermal property of CuS hollow nanocages (CuS HNCs) (Fig. 3). TA inhibited the oxidation of TMB and effectively captured the thermal holes generated by CuS HNCs under NIR irradiation, which suppressed the reaction system’s photothermal effect. The established method exhibited better selectivity and higher interference resistance from GA and AA.

**Fig. 4:**
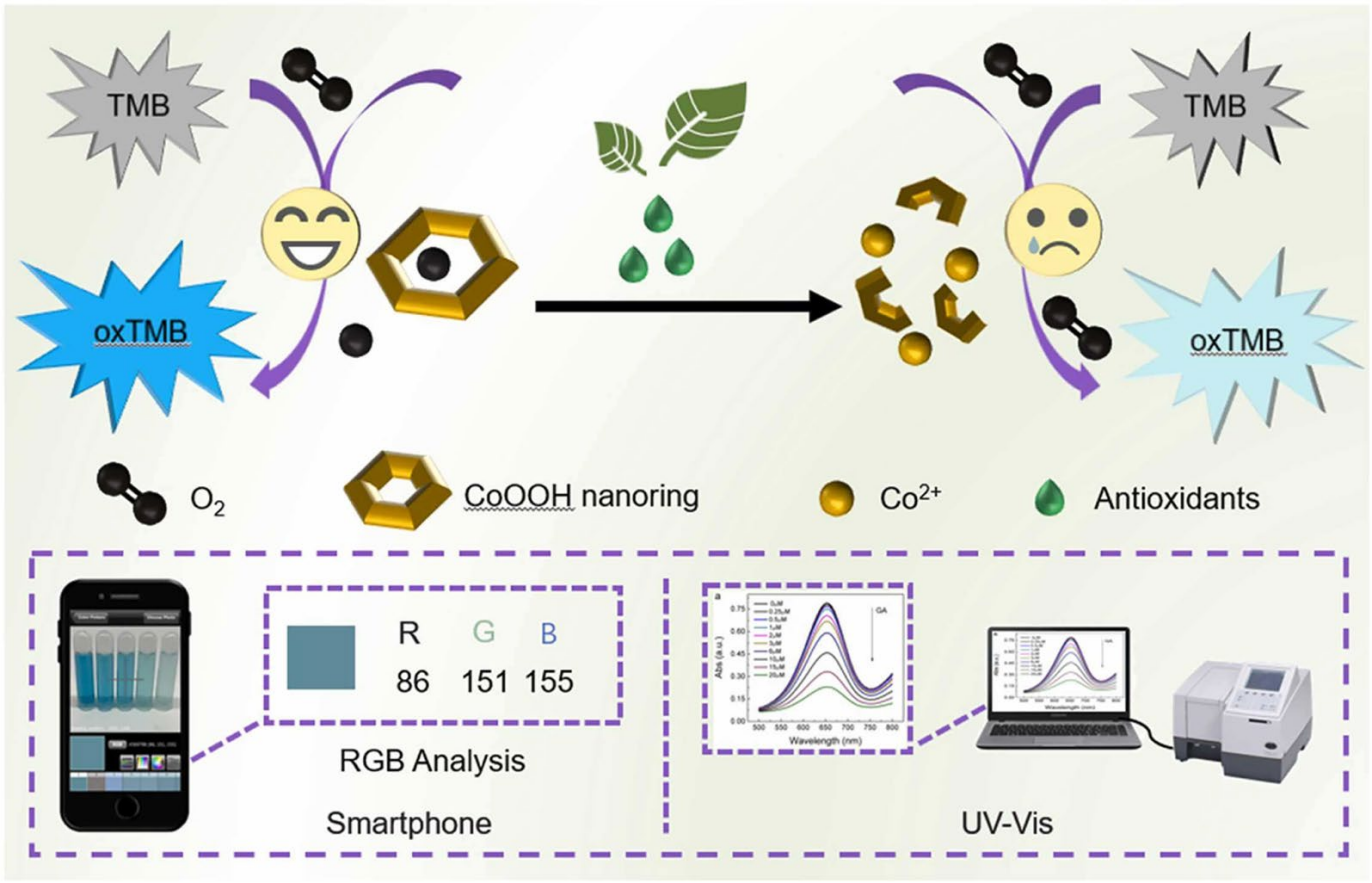
Schematic diagram of detecting antioxidants based on CoOOH nanoring. Reprinted with permission from [37]

However, most of the above methods failed to identify GA or TA with absolute specificity because of the interference of other antioxidants in the samples. Alternatively, similar methods were applied to detect total antioxidant capacity (TAC) in the actual samples. He et al. [36] designed a Pd–Pt-Ru nanozyme with good POD-like activity, which was used to detect ascorbic acid (AA) and H_2_O_2_ in the ranges of 2–12 µM and 5–40 mM with the LOD values of 1.13 µM and 2.79 mM, respectively. The method was applied in the evaluation of TAC of drinks (iced tea and green tea), foods (orange, lemon, and tomato), and herbs (*Cornus officinalis*, *Cynanchum otophyllum, Dioscorea bulbifera*, and Eriobotryae Folium). The results demonstrate that orange and *C. officinalis* have a higher TAC. Based on the OXD-like activity of cobalt oxyhydroxide (CoOOH) nanorings, Zhang et al. [37] developed a colorimetric sensor with smartphone assistance for the detection of antioxidants in green tea (Fig. 4). The detection mechanism is the decomposition of CoOOH nanorings into Co^2+^ after the addition of antioxidants, resulting in a decrease of catalytic activity. The established method exhibited high sensitivity with a LOD of 0.025 µM. Moreover, a smartphone can be used as a readout, and the content of total antioxidants in green tea was measured to be 2.55 µM, which is close to the result of Folin’s method. Meanwhile, Murilo et al. [38] synthesized the manganese dioxide/graphene quantum dot (MnO_2_/GQD) composites with excellent OXD-like activity, which was applied to detect the total antioxidants in fresh lemon juice, black tea, and mango juice, with recovery values of 95–105%. It is noteworthy that the system can differentiate different antioxidants by treating the obtained data through principal components analysis (PCA).

### Detection after sample pretreatment

Some active ingredients, such as quercetin and rutin, are insoluble in water, so long-time alcohol extractions are needed. During the preparation of real samples, Davoodi-Rad et al. [39] dried the vegetable samples at 60 °C for 4 h, then ground them into powder. A portion of the powder was mixed with methanol and stirred for 24 h, finally followed by filtration, washing, and dilution. Additionally, several plants, like *Datura stramonium*, contain diverse components. Therefore, the detection of specific components in them requires complex extraction processes. The preparation of *Datura* samples required drying and degreasing, which was first extracted with methanol and filtered, and then rotary evaporated to remove the solvent. Following ultrasonication, the samples were further extracted twice with dichloromethane (DCM). This process involved several separation steps, pH adjustments, and drying steps [40].

Quercetin, which is a type of naturally polyphenolic flavonoid compound, is one of the active ingredients in many frequently used CHMs and natural products, such as *Ginkgo biloba*, licorice, and onions. Quercetin has various properties, including antioxidant, anti-cancer, hypoglycemic, and liver-protective [41]. Cao et al. [42] synthesized the CeO_2_/Co_3_O_4_@N-doped hollow carbon microspheres (CeO_2_/Co_3_O_4_@NCH) through a self-template method, which exhibited excellent POD-like activity due to its larger surface area, pore-like structure, and OVs. Based on the reduction property of quercetin, a facile, fast, and cheap sensor was established to detect it with a linear range of 7–22 µM and a LOD of 1.19 µM. In addition, the sensor was applied to analyze quercetin in Yinxingye Dispersible Tablets, showing satisfactory recoveries. Moreover, some nanozymes based on LAC-like activity have also been developed for the quantitative analysis of quercetin. LAC is a copper-containing polyphenol oxidase that catalyzes polyphenols and polyamines to produce colored ortho-quinone [39, 43]. Davoodi-Rad et al. [39] synthesized the Cu-TA nanosheets (Cu-TA NSs) with LAC-like activity to detect quercetin in treated vegetable samples. Firstly, Cu-TA NSs can oxidize quercetin to generate ortho-quinone. Additionally, the addition of the surfactant cetyltrimethylammonium bromide (CTAB) reacted with quercetin by supramolecular interaction, further promoting the oxidation of quercetin. The developed method showed good selectivity to detect quercetin with a lower LOD of 0.064 µM. Then, it was used to detect the quercetin content in red onion, green pepper, and drill samples, and the results are in consistent with that of HPLC analysis.

Nanozyme-based detections for total polyphenols have also been developed. For instance, Rashtbari et al. [44] synthesized molybdenum trioxide nanoparticles through Argon cold plasma surface modification (Ar-MoO_3_NPs), which exhibited enhanced POD-like activity. The prepared Ar-MoO_3_NPs can oxidize non-fluorescent terephthalic acid into high-fluorescence emission compounds in the presence of H_2_O_2_. In contrast, polyphenols can cause aggregation of Ar-MoO_3_NPs and act as free radical scavengers, leading to the quenching of fluorescence. Therefore, a fluorescence method was developed to detect polyphenols with high specificity, which was successfully used to detect total polyphenolics in apple, orange, and grape samples.

**Fig. 5:**
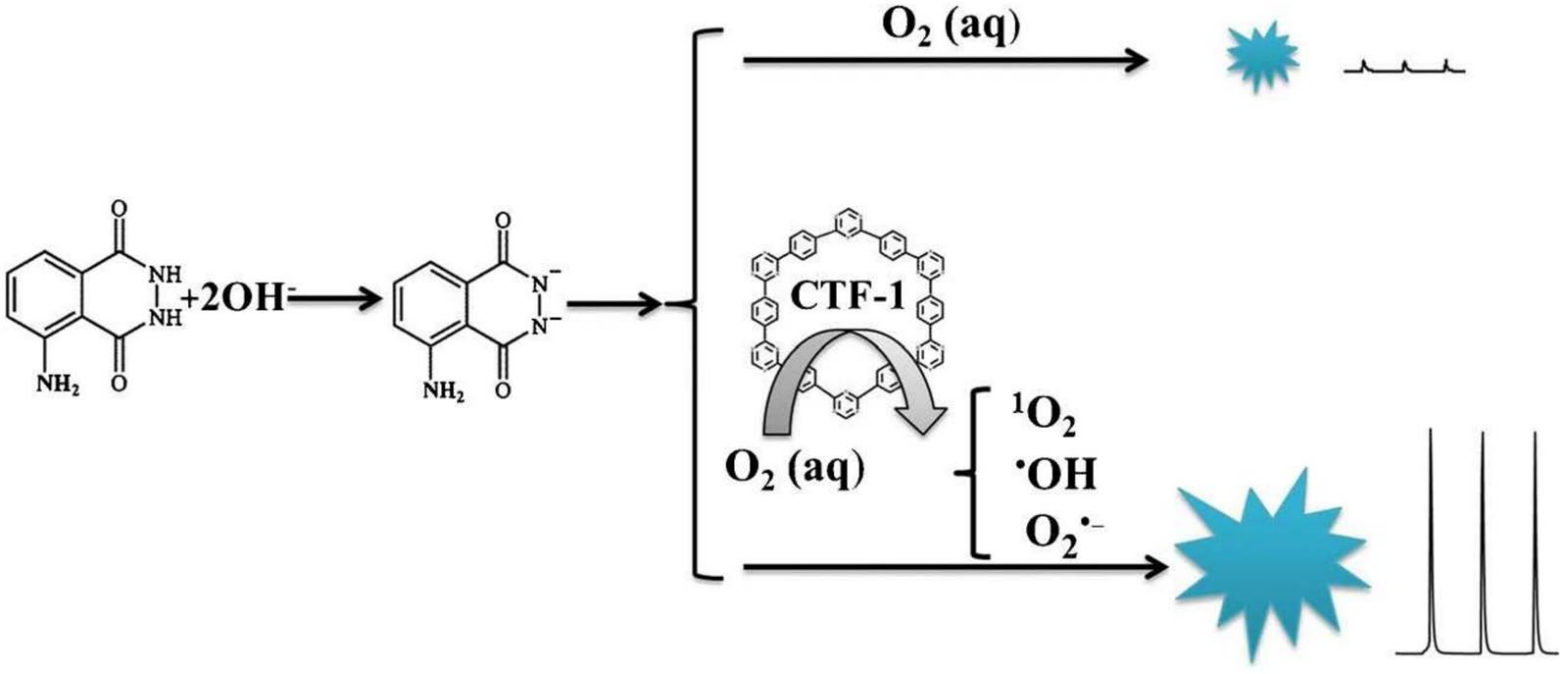
Schematic diagram of CL of luminol based on CTF-1. Reprinted with permission from [47]

Rutin, which has antioxidant, anticancer, vasoprotective, and neuroprotective properties [45], is a polyphenolic flavonoid compound and can be hydrolyzed to produce quercetin. Davoodi-Rad et al. [46] developed a colorimetric method for detecting rutin based on the LAC-like activity of Cu-guanosine nanorods (Cu-Guo NRs), which can oxidize rutin, resulting in a color change from light green to dark yellow. The established strategy has a broad linear range of 0.77–54.46 µM and a LOD of 0.114 µM. Then, it was successfully used to detect rutin in propolis dry extract and rutin-containing dietary supplement tablets, with contents of 9.42% and 18.38 mg per tablet, respectively. Covalent triazine framework (CTF) is a special class of COFs with a triazine ring in its structure. Based on the advantage of chemiluminescent (CL) detection with high sensitivity, Tan et al. [47] prepared a CTF-1 with OXD-like activity, which can oxidize luminol to produce intense CL in the presence of O_2_ (Fig. 5). Whereas the intensity of CL decreased with the increase of rutin concentration, therefore, a very sensitive CL method can be established for detecting rutin with a LOD of 0.015 µM. Compared with the results of HPLC, the developed CL method is reliable for detecting rutin both in tablets and treated Flos Sophorae Immaturus samples.

**Fig. 6:**
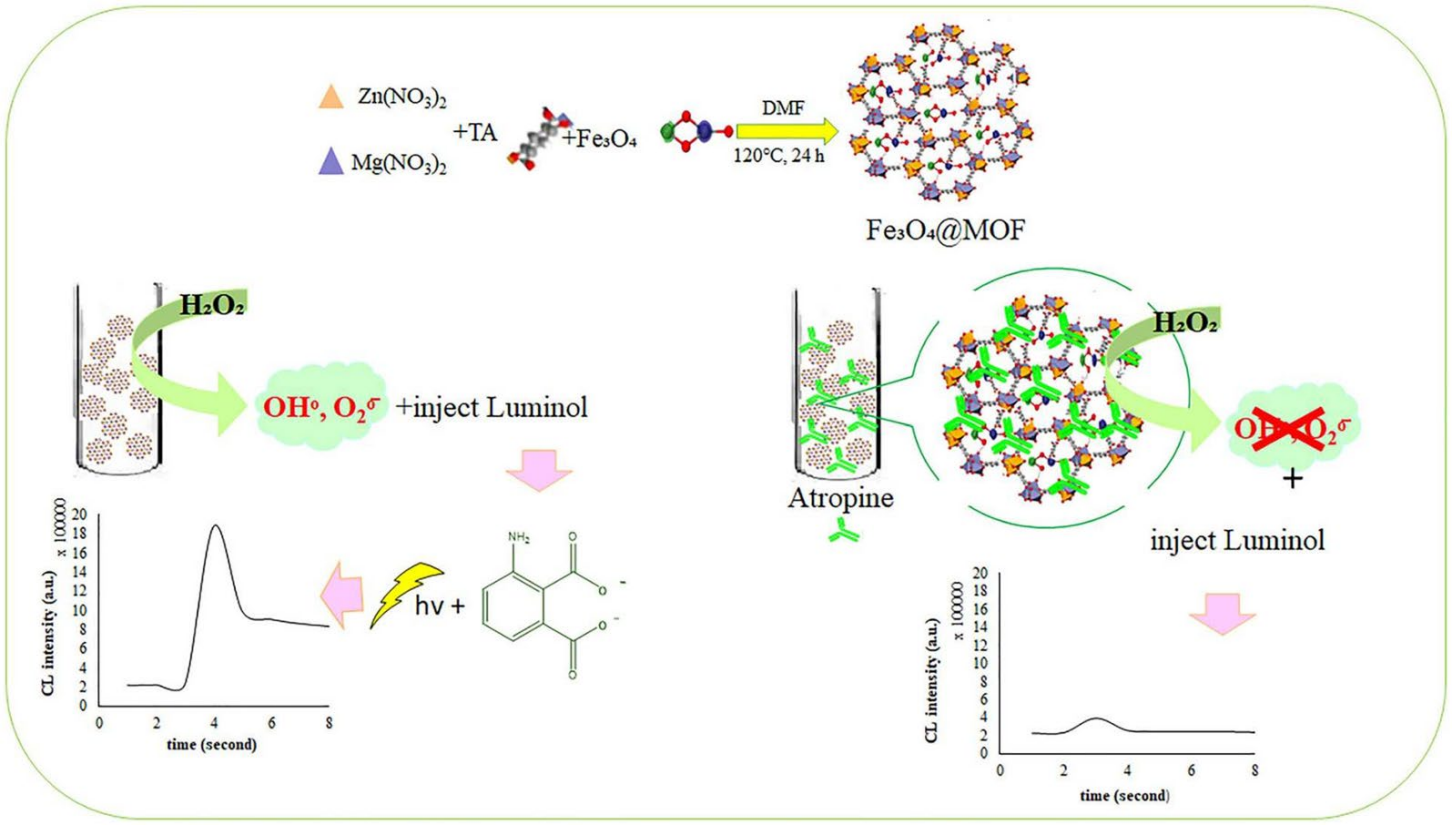
Schematic diagram of detecting atropine based on Fe_3_O_4_@MOFs. Reprinted with permission from [48]

Atropine is an alkaloid that can be used to dilate the pupil, alleviate spasms, and serve as an antidote to organophosphorus pesticides. *Datura* is a poisonous plant but contains abundant active chemicals, including phenolics, steroids, acyl sugars, amides, and alkaloids. Therefore, tedious sample pretreatment is necessary to detect atropine content in *Datura* plants. Mahmoudi et al. [40] synthesized a series of Fe_3_O_4_ and bimetal-organic framework Zn/Mg (Fe_3_O_4_@MOFs) composites for the detection of atropine extracted from two *Datura* samples through liquid–liquid extraction. The experimental results show that the Fe_3_O_4_@MOF/Dextrin composite exhibited the highest POD-like activity, which was primarily attributed to the cooperative interaction of dispersed Fe ions between Zn and Mg metals in the MOF and dextrin layers. In the presence of the material and H_2_O_2_, terephthalic acid was oxidized to 2-hydroxy terephthalic acid, emitting fluorescence at 425 nm. This oxidation process can be inhibited by atropine, allowing the fluorescence detection of atropine with the LOD value of 2.27 μg/L. To further improve the sensitivity of atropine, the group [48] developed a CL method to detect atropine based on the Fe_3_O_4_@MOF composite, which can oxidize luminol, producing high-intensity CL (Fig. 6). While atropine can bind with the Fe_3_O_4_@MOF composite, leading to a significant reduction of CL intensity. Compared with the previous method, the sensitivity was considerably improved and the LOD was as low as 0.02 μg/L.

### Specific recognition strategy

**Fig. 7:**
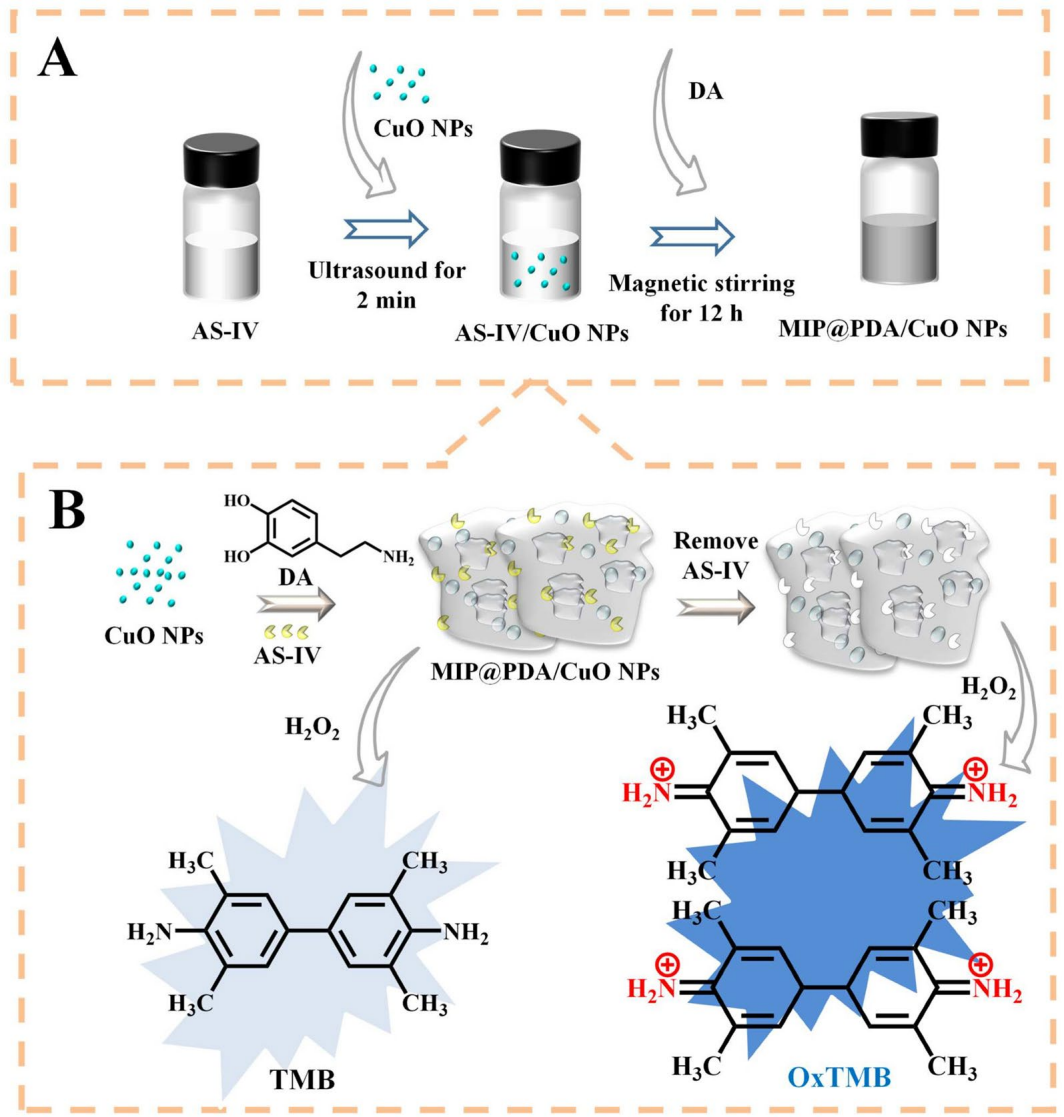
Schematic diagram of preparing MIP@PDA/CuO NPs (**A**) and detecting AS-IV (**B**). Reprinted with permission from [51]

Molecularly imprinted polymers (MIPs) are formed by polymerizing monomers in the presence of a template molecule. After removing the template molecule, MIPs can be used to bind template molecule specifically, similar to the interaction between an antibody and an antigen [49, 50]. Therefore, MIP can extract template molecules from complicated samples and shield interference from other substances, improving the assay selectivity [51]. As one of the main active ingredients in Huangqi (*Astragalus membranaceus*), Astragaloside-IV (AS-IV) has many pharmacological activities, including enhancing immunity, antivirus, anti-stress, antifibrosis, and protecting the heart [52, 53]. The AS-IV is usually detected by HPLC in combination with other methods due to its weak ultraviolet absorption, such as pulsed amperometric detection and evaporative light scattering detection (ELSD) [53, 54]. Chen et al. [51] innovatively combined MIP with CuO nanoparticles (CuO NPs) and polydopamine (PDA) to synthesize MIP@PDA/CuO NPs with POD-like activity for the detection of AS-IV. AS-IV can specifically bind to the surface imprinted cavity to prevent the entry of H_2_O_2_ and TMB, inhibiting the catalytic process (Fig. 7). Eventually, the established new colorimetric method for the detection of AS-IV showed high selectivity and the linear range was 0.000341–1.024 mg/mL with a LOD of 0.000991 mg/mL. Additionally, it was applied to detect the content of AS-IV in Huangqi Granules and Ganweikang Tablets, and the results are similar to that measured by HPLC-ELSD.

**Fig. 8:**
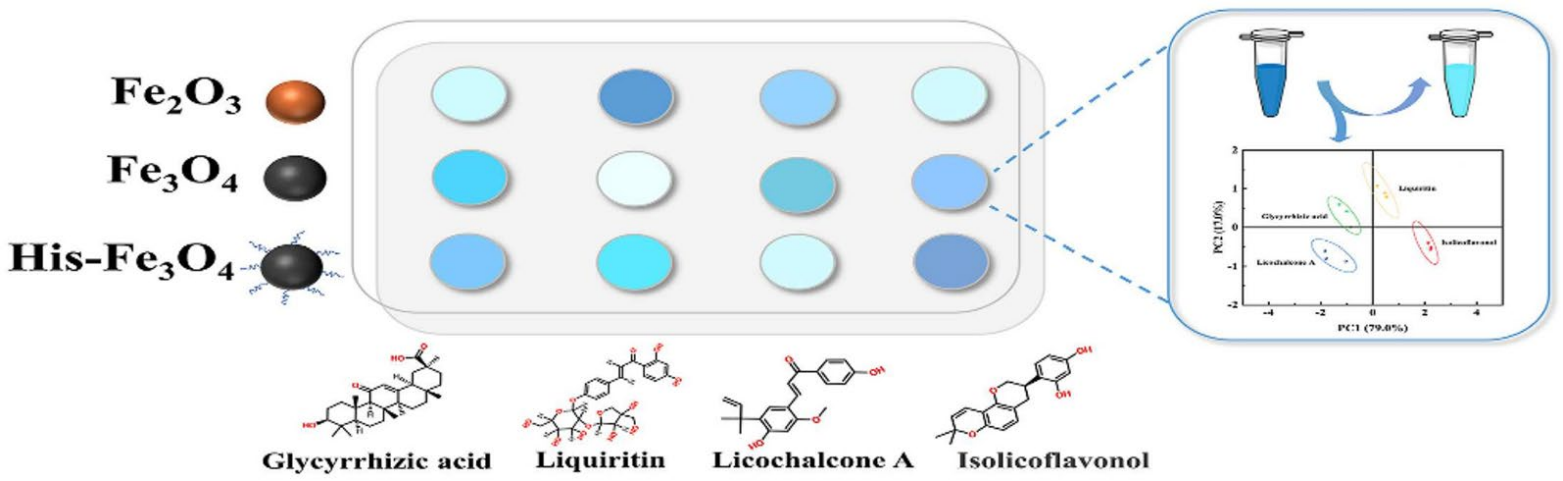
Schematic diagram of detecting four licorice active substances based on the colorimetric sensor array. Reprinted with permission from [56]

Licorice (*Glycyrrhiza uralensis*) is a CHM with diverse functions, such as anti-inflammatory and detoxification. Among licorice active ingredients, liquiritin and glycyrrhizic acid are the indicators for authenticating licorice, while licochalcone A and isolicoflavonol are the indicators for evaluating its quality. Thus, the simultaneous detection of these four active substances is of great significance. The sensor array consists of a series of cross-response sensing units rather than a specific receptor, which can detect and discriminate structurally similar components or complex mixtures through pattern recognition [55]. Based on three iron oxide nanozymes (Fe_2_O_3_, Fe_3_O_4_, and histidine (His)-Fe_3_O_4_) with POD-like activity, Yuan et al. [56] constructed a colorimetric sensor array for the detection of four licorice active substances (Fig. 8). Different active ingredients inhibited the catalytic activity of different iron oxides to various degrees, and the developed colorimetric sensor successfully identified and distinguished the four licorice active substances in real licorice samples in the concentration range of 1–200 µM.

Although nanozyme-based detection methods have the advantages of simplicity, rapidity, and high sensitivity, there were limited number of methods have been developed for the detection of phytochemicals in natural products using nanozymes. Furthermore, due to the complexity of real samples, there are still difficulties in achieving specific detection, which requires sample pretreatment or combining with special methods such as MIP. Therefore, the design and preparation of nanozymes with high selectivity to solve the problem of complex sample matrix in phytochemical analysis remain in the exploratory stage.

## Detection of hazardous substances

### Detection of heavy metal ions

Due to their bioaccumulation properties, heavy metal ions can reach very high levels through the diet, thereby harming human health [8]. Common heavy metal elements include arsenic (As), lead (Pb), mercury (Hg), copper (Cu), chromium (Cr), cadmium (Cd), iron (Fe), etc. [57]. As shown in Table 3, there are several studies on the detection of As and Pb in plants using nanozymes.

**Table 3:** Summary of detection of heavy metal ions in plants based on nanozymes

Nanozyme/activity	Analyte	Method	Sample	LOD(µM)	Linear range(µM)	Refs.
Ce(IV)-ATP-Tris CPNs/OXD	As^5+^	Colorimetric	Rice	0.44 μg/L	0.67–2666.67 μg/L	[60]
ACP/hemin@Zn-MOF/POD	As^5+^	Ratio fluorescence	Rice samples	1.05 μg/L	3.33–300.00 μg/L	[58]
Octahedral Mn_3_O_4_ NPs/OXD	As^3+^	Colorimetric	Wheat and water samples	1.32 μg/L	5–100 μg/L	[59]
Fe, NA-CDs/PB/POD	Pb^2+^	Colorimetric/SERS	Barley Yellow, *Salvia miltiorrhiza*, *Astragalus membranaceus*, and pomegranate peel	0.015 × 10^–3^/0.024 × 10^–3^	0.03 × 10^–3^–3 × 10^–3^	[64]
WS_2_ nanosheets/POD	Pb^2+^	Colorimetric	Tap water, soil, wheat, and fish serum	0.012 × 10^–3^	0.015 × 10^–3^–0.24 × 10^–3^	[66]
porph@MOF/POD	Pb^2+^	Electrochemistry	Chinese cabbage and spinach	4.8 × 10^–9^	10 × 10^–9^–0.1	[65]
SACu-C-N/OXD	Hg^2+^	Colorimetric	Water, sea bass, cabbage, and honey	0.85 × 10^–3^	0.001–20	[75]
CTF/POD	Cu^2+^	Colorimetric	Eggplants and Chinese water chestnuts	1.25 × 10^–3^	15.75 × 10^–3^–1.26 × 10^3^	[80]
CuO NP-POM/GPx	Fe^2+^/AA	Fluorescence	Spinach and dried	0.008/0.015	0.01–100/0.02–500	[148]
p-β-CD@Pr_6_O_11_/OXD	Fe^2+^/Cys	Colorimetric	Spinach juice, black fungus, pork, and pork liver/Water, FBS, and Cys capsules	0.098/0.01	0.1–14/0.01–5	[149]
SACe-N-C/OXD	Fe^3+^/Cr^6+^	Colorimetric	Wheat, peach, teas, celery, spinach, and chickens	34.72/93.65 ng/mL	0.25–1.5/0.5–5 mg/mL	[76]
AuNCs/POD	Hg^2+^/Cu^2+^/Co^2+^/Cd^2+^/Pb^2+^	Colorimetric sensor array	Water, *Lonicera japonica*, and *Chrysanthemum morifolium*	0.05/0.2/0.05/2.5/1	0.05–0.8/0.2–0.8/0.05–0.8/2.5–25/1–10	[82]

POD, peroxidase; OXD, oxidase; ATP, adenosine triphosphate; Tris, tris hydroxymethyl aminomethane; CPNs, coordination polymer nanoparticles; ACP/hemin@Zn-MOF, acid phosphatase and hemin loaded Zn-based metal–organic framework nanosheets; POD, peroxidase; NPs, nanoparticles; Fe, NA-CDs, Fe doped norepinephrine-based carbon dots; PB, Prussian blue; SERS, surface-enhanced Raman scattering; MOF, metal–organic framework; SACu-C-N, single-atom Cu-C-N; SACe-N-C, single atom Ce-N-C; AuNCs, gold nanoclusters; CTF, covalent triazine frameworks; CuO NP-POM, polyoxometalate (POM) decorated with copper oxide nanoparticles (CuO NPs); AA, ascorbic acid; GPx, glutathione peroxidase; p-β-CD@Pr_6_O_11_, poly-β-cyclodextrin strengthen praseodymium oxide (Pr_6_O_11_) porous oxidase mimic; Cys, cysteine; FBS, fetal bovine serum

#### Arsenic ion

**Fig. 9:**
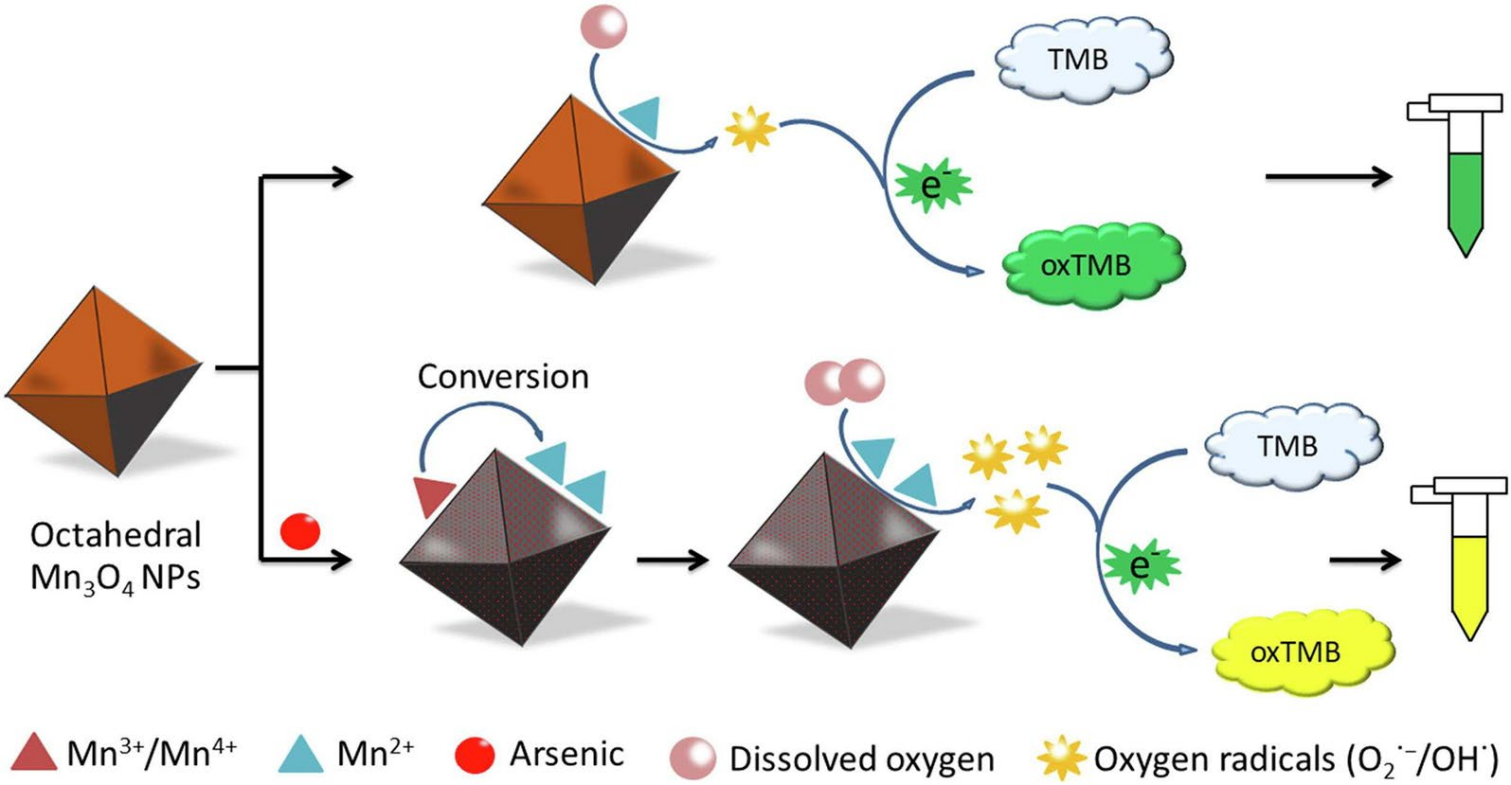
Schematic diagram of detecting As (III) based on octahedral Mn_3_O_4_ NPs. Reprinted with permission from [59]

Compared with organic arsenic, inorganic arsenic exhibits higher toxicity. Excessive intake of it can cause skin and respiratory diseases, nerve poisoning, organ failure, and even cancer, which may be resulted from its interaction with enzymes in the human body and excess generation of reactive oxygen species (ROS) [58–61]. Inorganic arsenic includes arsenic trivalent (As(III)), arsenic pentavalent (As(V)), and elemental arsenic, while As(III) is more toxic than As(V) as it can bind to sulfhydryl groups with higher affinity, inhibiting the activity of various proteins [59, 61]. Wang et al. [59] developed a colorimetric method to detect As(III) (Fig. 9). They synthesized different shapes of Mn_3_O_4_ NPs with OXD-like activity, while the octahedral one possessed the strongest As(III) adsorption capacity. Furthermore, arsenic adsorption made Mn^4+^/Mn^3+^ reduced to Mn^2+^, which can catalyze O_2_ to produce oxygen radicals, further oxidizing TMB with the solution turning to yellow color. Based on As(III)-adsorption enhancing the catalytic activity of octahedral Mn_3_O_4_ NPs, a colorimetric method was established and achieved the detection of As(III) in the wheat sample with a LOD of 1.32 μg/L.

As(V) can inhibit the catalytic activity of acid phosphatase (ACP), which can catalyze the hydrolysis of ascorbic acid 2-phosphate (AAP) to produce AA. Therefore, several studies have utilized nanozymes and ACP to design enzyme-cascade reactions for As(V) detection. Xu et al. [58] prepared an ACP and hemin-loaded multifunctional Zn-based metal–organic framework (ACP/hemin@Zn-MOF) for the detection of As(V). Hemin exhibited POD-like activity, which can catalyze the oxidation of *o*-phenylenediamine (OPD) to form a fluorescent product (564 nm) and weaken its intrinsic fluorescence (452 nm) owing to the inner filter effect. After the addition of AAP, the generated AA will competitively suppress the oxidation of OPD, causing a decrease in the fluorescence intensity at 564 nm and a recovered fluorescence at 452 nm. The inhibitory effect of As(V) on ACP enabled the fluorescence signal to be reversed again, realizing a ratio fluorescence detection of As(V) with a linear range of 3.33–300.00 μg/L and a LOD of 1.05 μg/L. Moreover, the method was successfully applied in the analysis of As(V) and total arsenic in rice samples, with recovery rates ranging from 95 to 105%. Similarly, Wang et al. [60] developed a colorimetric method to detect As(V) utilizing the OXD-like activity of Ce(IV) coordination polymer nanoparticles. With the addition of ACP and AAP, the produced AA can not only restrain the oxidation of TMB but also reduce Ce^4+^ to Ce^2+^, inhibiting the enzyme-like activity of the material. Therefore, As(V) can be detected by inhibiting ACP and restoring the TMB color reaction. The method displays a high sensitivity and was used to analyze As(V) in rice samples.

#### Lead ion

**Fig. 10:**
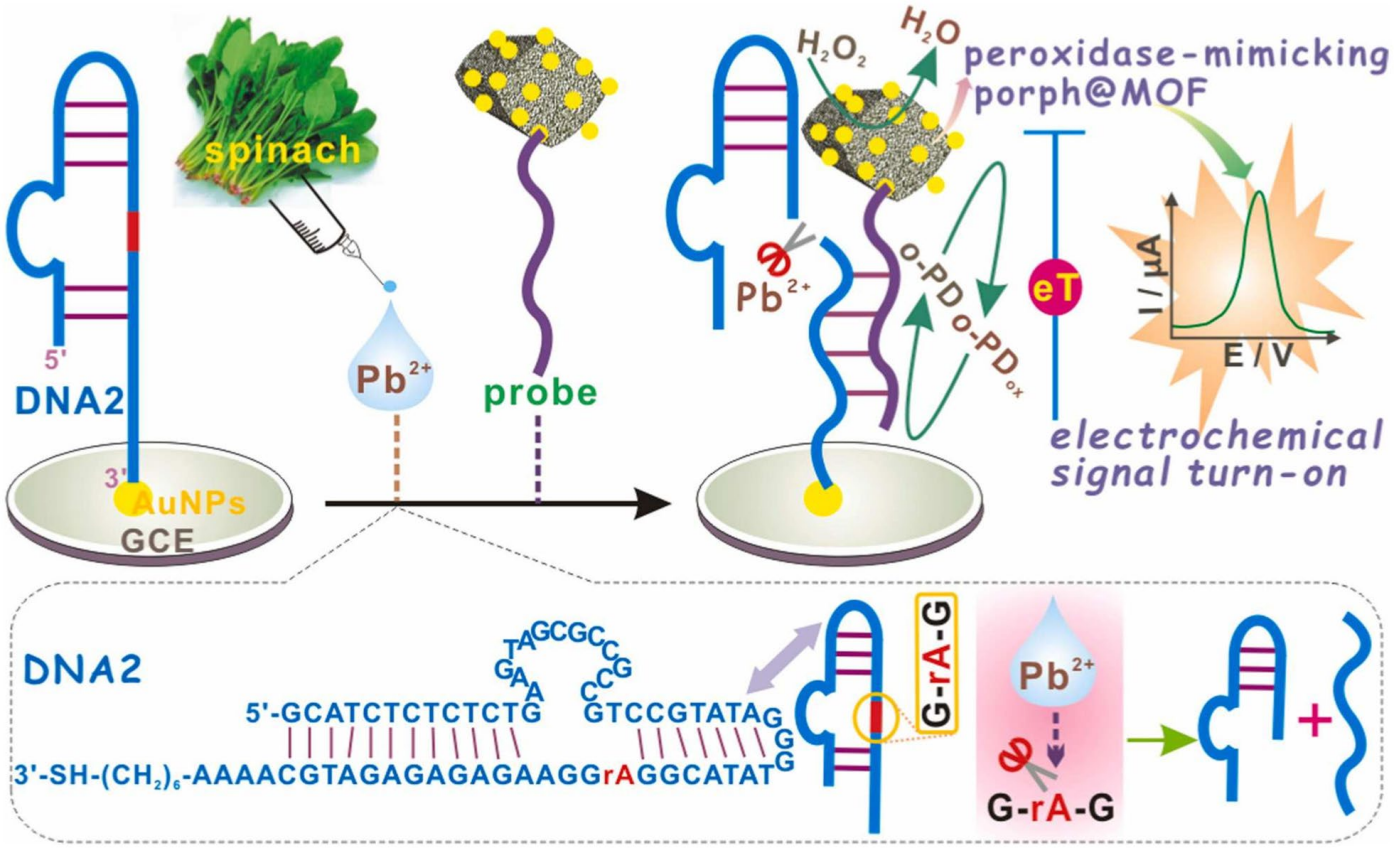
Schematic diagram of detecting Pb.^2+^ based on DNAzyme and porph@MOF. Reprinted with permission from [65]

Pb is the second most toxic heavy metal after As with bioaccumulation and persistence [62–66]. Low doses of Pb^2+^ have an impact on the physical and mental health of infants and young children, causing developmental disorders, brain damage, psychiatric disorders, etc. [67]. Tang et al. [66] synthesized the layered WS_2_ nanosheets with POD-like activity through a simple ultrasonic stripping method, which was employed to detect Pb^2+^ in wheat samples. Pb^2+^ blocked the electron transfer between WS_2_ and H_2_O_2_, and then prevented the oxidation of TMB, resulting in a significant decrease of absorbance at 650 nm. For Pb^2+^ detection, the linear range of the method was 0.015–0.24 nM with a low LOD of 0.012 nM. Using Pb^2+^-dependent receptors (e.g. DNAzyme) is a good option to achieve a high selective detection. Si et al. [65] developed an electrochemical method to monitor Pb^2+^ in vegetable samples based on a porphyrin-functionalized metal–organic framework (porph@MOF) and Pb^2+^-dependent DNAzyme. As shown in Fig. 10, DNA2 was immobilized on an AuNPs-modified glassy carbon electrode via the Au–S bond. It can be specifically cleaved by Pb^2+^ to generate a short DNA2 fragment, which was further hybridized with porph@MOF-DNA1 through base pairing. Subsequently, the porph@MOF with POD-like activity oxidated OPD in the presence of H_2_O_2_, producing the electrochemical signal. The established method exhibited excellent selectivity and high sensitivity with a LOD of 5 pM. However, although cleavage and hybridization of DNA2 can be finished in one step, the long incubation time (80 min) did not fulfill the requirement of rapid detection. Colorimetric and surface-enhanced Raman spectroscopy (SERS) are commonly regarded as rapid analytical methods [68]. Gold nanoparticles (AuNPs) are widely studied for their enzyme-like and SERS properties [69, 70]. Furthermore, several studies have substantiated that carbon dots (CDs) facilitate improving SERS signals and catalytic activity of AuNPs [71–73]. For example, Cui et al. [64] prepared the Fe-doped norepinephrine-based CDs through a one-step microwave digestion method, which were self-assembled with Prussian blue (PB) to obtain Fe, NA-CDs/PB with POD-like activity. They also utilized the reducing property of CDs to synthesize the AuNPs. Based on the inhibition of Pb^2+^ on the POD-like activity of Fe, NA-CDs/PB and AuNPs, the SERS and colorimetric dual-mode sensor was constructed with the LODs of 0.024 nM and 0.015 nM, respectively. Finally, the sensor was successfully applied to detect Pb^2+^ in *Salvia miltiorrhiza*, *Astragalus membranaceus*, Barley Yellow, and pomegranate peel with good recovery of 90.4–108.9% and RSD of 2.6–4.7%.

#### Other ions

Ingestion of inorganic mercury may cause neurological symptoms (including mental retardation, vision and hearing loss, language disorders, and memory loss), as well as cognitive and motion disorders, etc. [74, 75]. Recently, single-atom nanozymes (SAzymes) with ultra-high atomic utilization, excellent stability, and remarkable catalytic activity have been continuously studied [75–77]. Ge et al. [75] developed a novel colorimetric strategy for Hg^2+^ assay using cysteine and single-atom Cu-C-N nanozymes (SACu-C-N). The prepared SACu-C-N exhibited OXD-like activity, catalyzing the oxidation of TMB to blue ox-TMB. However, the ox-TMB was reduced after the introduction of cysteine. Since Hg^2+^ possesses a strong affinity for thiol groups of cysteine, it can turn the solution to blue color again. Therefore, a simple, sensitive, and selective colorimetric method was established and applied in the analysis of Hg^2+^ in cabbage samples.

Cu is an essential trace element and is influential in the metabolic process as a cofactor or structural component of many natural enzymes [78–80]. However, excess Cu^2+^ suppresses the activity of some essential enzymes, causing serious side effects, such as neurodegenerative disease, liver damage, and even cancer [79, 81]. In addition, high levels of Cu also damage photosynthesis and then inhibit plant growth [78]. Xiong et al. [80] synthesized a CTF through a simple and rapid microwave-enhanced high-temperature ionothermal method. Interestingly, CTF possessed weak POD-like activity, but Cu^2+^ can act as the active catalytic center and a bridge for electronic transfers between the substrate and CTF, resulting in enhanced catalytic activity. The LOD value of the developed method was 1.25 nM, and was applied in the quantification of Cu^2+^ in Chinese water chestnuts and eggplants, with recoveries of 96.0–105.0%.

Most of the sensors can detect only a single heavy metal, and the simultaneous detection of multiple heavy metal ions is still a challenge. Song et al. [76] designed a time-resolved sensor to detect Cr^6+^ and Fe^3+^ based on their difference in enhancing the single-atom Ce–N–C nanozyme’s OXD-like activity. In the presence of Fe^3+^ and Cr^6+^ alone, the solution turned blue after 30 s and 60 s, respectively, while the former faded after 5 min. The solution turned blue in 30 s and did not fade when both of them were presented. Therefore, the constructed sensor was feasible for the simultaneous detection of Fe^3+^ and Cr^6+^ in actual samples. By utilizing gold nanoclusters (AuNCs) as sensing elements, Li et al. [82] developed a colorimetric sensor array for identifying five heavy metal ions (Hg^2+^, Pb^2+^, Cu^2+^, Cd^2+^, and Co^2+^) at a concentration down to 0.5 μM, which was successfully used to recognize multiple heavy metal ions in *Lonicera japonica* and *Chrysanthemum morifolium* samples.

### Detection of mycotoxins

Mycotoxins are secondary metabolites generated by filamentous fungi and are extensively found in maize, wheat, rice, peanuts, and other cereals, which include aflatoxins, ochratoxins, fumonisins, zearalenone [14, 83], etc. Even at low concentrations, they are nephrotoxic, immunotoxic, teratogenic, mutagenic, and carcinogenic [84, 85]. According to the Chinese Pharmacopoeia, the total aflatoxin content in Chinese medicines should not exceed 10 µg/kg, and zearalenone should not exceed 500 µg/kg [86]. At present, a number of nanozyme-based immunoassays have been reported for detecting mycotoxins (Table 4).

**Table 4:** Summary of detection of mycotoxins in plants based on nanozymes

Nanozyme/activity	Analyte	Assay format	Method	Sample	LOD (ng/mL)	Linear range (ng/mL)	Ref
MnO_2_ NSs/OXD	Fumonisin B1	NLISA	Colorimetric	Corn and wheat	0.63	1.17–20.74	[150]
PCu/POD	*Aspergillus flavus*	LFIA	Colorimetric/photothermal	Peanut and corn	0.45/0.22	1–1 × 10^5^	[96]
MnO_2_ NSs/OXD	Aflatoxin B1	LFIA	Colorimetric	Corn	0.015	0.01–150	[95]
CuCo@PDA/POD	Aflatoxin B1	Apt-LFA	Colorimetric	Peanut, wheat, and corn	2.2 × 10^–3^	0.01–500	[97]
Cu_2_O@Au NCs/POD	Aflatoxin B1	Aptasensor	SERS	Peanut	0.007	0.001–100	[93]
L-Cys-FeNiNPs/POD	Aflatoxin B1	Aptasensor	Colorimetric	Corn and millet	36.57	120–2000	[151]
Au/Ni-Co LDH NCs/POD	Aflatoxin B1	Aptasensor	Electrochemical/colorimetric	Corn	0.071 × 10^–3^/18.6 × 10^–3^	0.0002–100/0.05–100	[152]
Fe-N-C SAzymes/POD	Aflatoxin B1	NLISA	Colorimetric	Peanut	3.3 × 10^–3^	0.0084–0.358	[77]
Pt-CN/POD	Aflatoxin B1	ELISA	Colorimetric/photothermal	Peanut	0.22 × 10^–3^/0.76 × 10^–3^	0.001–10	[101]
PS@Pt-Pd/OXD	Aflatoxin B1	NLISA	Colorimetric	Peanut	5.52 × 10^–3^	0.01–0.104	[91]
MnO_2_ NSs/OXD	Aflatoxin B1	Immunosensor	Colorimetric	Peanut	6.5 × 10^–3^	0.05–150	[87]
MNPs/PBNPs/POD	Aflatoxin B1	NAISA	Photothermal/colorimetric/fluorescence	Vinegar, wine, and peanut	3.42 × 10^–3^/15.07 × 10^–6^/0.54 × 10^–6^	10^–2^–100/10^–4^–100/10^–5^–100	[90]
m-SAP/POD	Aflatoxin B1	NLASA	Colorimetric	Peanut	5 × 10^–3^	0.01–1000	[153]
Pt@PCN-222/OXD	Aflatoxin B1	–	Colorimetric	Peanut and corn	0.074 × 10^6^	0.1–10	[89]
CHNPs/OXD	ALP Aflatoxin B1	Immunosensor	Colorimetric	Peanut	0.003 U/L 0.73 × 10^–3^	− 0.001–20	[99]
Octahedral Cu_2_O NPs/POD	Ochratoxin A	NLISA	Colorimetric	Millet	470	1 × 10^6^–5 × 10^6^	[110]
Co(OH)_2_ nanocages/OXD	Ochratoxin A	NLISA	Colorimetric	Corn and water samples	260	500–5 × 10^6^	[102]
AuNPs/POD	Ochratoxin A	Aptasensor	Colorimetric	Oats, corn, soybeans, rice, and glutinous rice	6.20 nM	0.01–0.6 µM	[103]
Cu@Fe-NC/POD	Ochratoxin A	NLISA	Colorimetric/ratio fluorescence	Corn and millet	790/520	10^3^–10^4^	[105]
Co/NCNT/OXD	Ochratoxin A	NLISA	Colorimetric/fluorescence	Corn and millet	210/170	1–10^4^	[106]
Pd-Pt NRs/POD	Ochratoxin A	NLASA	Colorimetric/SERS	Red wine and grape	0.097/0.042 nM	0.1–40 nM	[109]
AuAg NCs-SPCN/POD	Ochratoxin A	NLISA	Fluorescence/colorimetric	Red wine, wheat flour, and corn	155/213	10^3^–10^7^	[108]
CPNs(IV)/OXD	Ochratoxin A	ELISA	Fluorescence/colorimetric	Corn	0.404/0.962	4.69–37.50/14–300	[100]
Cu_2_O@Fe(OH)_3_ yolk-shell nanocages/POD	Ochratoxin A	NLISA	Ratio fluorescence/colorimetric	Millet and lake water	560/830	10^3^–10^7^	[107]
TiO_2_-PCA/OXD	Zearalenone	Aptasensor	Colorimetric	Corn and wheat	8.7 × 10^–3^	0.01–2	[154]
Ti_3_C_2_T_x_/AuNPs nanocomposite/POD	Zearalenone	NLISA	Colorimetric/photothermal	Rice, oats, and corn	0.15 × 10^–3^/0.48 × 10^–3^	500–5 × 10^5^	[112]
AuPt NPs/POD	Zearalenone	Aptasensor	Colorimetric	Corn and wheat	0.6979	1–250	[113]
Pt@AuNF/POD	Zearalenone	LFIA	Colorimetric	Corn	0.052	0.052–7.28	[111]
AuNPs/POD	Zearalenone	Aptasensor	Colorimetric	Corn and corn oil	10	10–250	[114]
ssDNA-g-C_3_N_4_ NSs/POD	Ochratoxin A/fumonisin B1/aflatoxin B2/zearalenone/aflatoxin M1	–	Colorimetric sensor array	Corn	0.001 µM	–	[155]

SERS, surface-enhanced Raman spectroscopy; NSs, nanosheets; POD, peroxidase; OXD, oxidase; NLISA, nanozyme-linked immunosorbent assay; NAISA, nanozyme and aptamer-based immunosorbent assay; NLASA, nanozyme-linked apta-sorbent assay LFIA, lateral flow immunoassay; ELISA, Enzyme-linked immunosorbent assay; Cu_2_O@Au NCs, Cu_2_O@Au nanocubes; PS@Pt–Pd, platinum and palladium bimetallic nanozyme modified polystyrene (PS) microspheres; Apt-LFA, aptamer-mediated lateral flow assay; Cu@Fe-NC, CuFe-bimetal coordinated N-doped carbon; Co/NCNT, Co nanoparticle/N-doped carbon nanotubes; L-Cys-FeNiNPs, L-cysteine-functionalized FeNi bimetallic nanoparticles; SAzymes, single-atom nanozymes; PCA, 3, 4-dihydroxybenzoic acid; AuNPs, gold nanoparticles; Pt-CN, Pt supported on nitrogen-doped carbon amorphous; AuAg NCs, Au–Ag nanoclusters; SPCN, S, P co-doped graphitic carbon nitride (g-C_3_N_4_) nanosheets; CHNPs, copper hexacyanoferrate nanoparticles; ALP, alkaline phosphatase; Pd-Pt NRs, Pd-Pt bimetallic nanocrystals; PCu, Cu-anchored inherent photothermal polydopamine (PDA); Pt@AuNF, platinum gold nanoflower; MNPs/PBNPs, magnetic nanoparticles/Prussian blue nanoparticles; m-SAP, AuPt nanoparticles loaded mesoporous SiO_2_ nanospheres; Pt@PCN-222, Pt nanoparticles loaded zirconium-porphyrin-MOF; Au/Ni-Co LDH NCs, Au nanoparticles anchored Ni-Co layered double hydroxides nanocages; CPNs(IV), cerium-based nanoparticles; ssDNA, single-stranded DNA

#### Aflatoxin b1

Aflatoxins include aflatoxin B1, B2, G1, and G2 [87]. Among them, aflatoxin B1 (AFB1) is the most toxic one with potent hepatocarcinogens, which was classified as a Group 1 carcinogen as early as 2002 for it can induce formatting DNA adducts, leading to hepatoma [88–90]. Apart from this, AFB1 is also associated with malnutrition, growth impairment, and immune inhibition [91–93]. Lateral flow immunoassay (LFIA) is a real-time analysis method on paper-based equipment. The principle is mainly based on the competitive binding of the target analyte and the fixed antigen on the detection line to the antibody [94]. Owing to its simplicity, rapidity, and low cost, LFIA has become an attractive immunoassay for AFB1 analysis. However, the limited sensitivity hinders LFIA's practical applications [95–97]. As mentioned, nanozymes can catalyze the formation of chromogenic substrates for signal amplification to improve the sensitivity of a detection method. Cai et al. [95] prepared MnO_2_ nanosheets with excellent OXD-like activity as signal labels conjugated with antibodies to detect AFB1. Using MnO_2_ catalyzing TMB to produce clear color signals, the method achieved sensitive detection of AFB1 with a LOD of 15 pg/mL and a wide linear range of 0.01–150 ng/mL. Compared to antibodies, aptamers are more stable and flexible in labeling. Therefore, aptamer-mediated LFIA is a promising approach to realize a highly sensitive detection. Zhu et al. [97] designed a PDA-modified nanozyme (CuCo@PDA) with abundant amide groups that can be coupled to AFB1 aptamers via a condensation reaction. Based on the POD-like activity of CuCo@PDA, a reliable and ultrasensitive method combined with a smartphone was established for AFB1 detection with a LOD of 2.2 pg/mL. Moreover, it was successfully applied to detect AFB1 in the peanut, corn, and wheat samples with different contamination levels.

The enzyme-linked immunosorbent assay (ELISA) is a widely used immunoassay. In a typical ELISA assay, antigens (analytes) first bind to antibodies immobilized on a well plate, then forming an antibody-antigen–antibody sandwich with enzyme-labeled antibodies (commonly HRP). After washing steps, HRP catalyzes the added substrate, resulting in a color change [98]. Likewise, the combination of nanozymes can effectively improve the relatively low sensitivity of ELISA [77, 99]. Guo et al. [77] developed a nanozyme-linked immunosorbent assay (NLISA) by utilizing Fe–N–C SAzymes to replace HRP for quantitative detection of AFB1 in peanut samples. Unlike the traditional antibody-antigen–antibody “sandwich” type of detection mechanism, the method immobilized antigens on the well plate, achieving the rapid and sensitive detection of AFB1 with a LOD of 3.3 pg/mL. The coupling of nanozymes with bio-enzymes induces a dual signal amplification through an enzyme cascade to further increase the sensitivity. Lai et al. [99] found that copper hexacyanoferrate nanoparticles (CHNPs) with OXD-like activity can be rapidly produced by simply mixing potassium hexacyanoferrate(III) (K_3_[Fe(CN)_6_]) with Cu(II). However, AA produced by the hydrolysis of ALP on ascorbic acid 2-phosphate (AAP) can reduce Fe (III) to Fe (II) and then inhibit the formation of CHNPs. Therefore, employing AuNPs coupled to ALP as enzyme labels in ELISA and integrating with the production process of CHNPs, a highly sensitive colorimetric immunoassay for the determination of AFB1 was constructed with a LOD of 0.73 pg/mL. Finally, the developed method was used to detect AFB1 in spiked and naturally contaminated peanut samples.

**Fig. 11:**
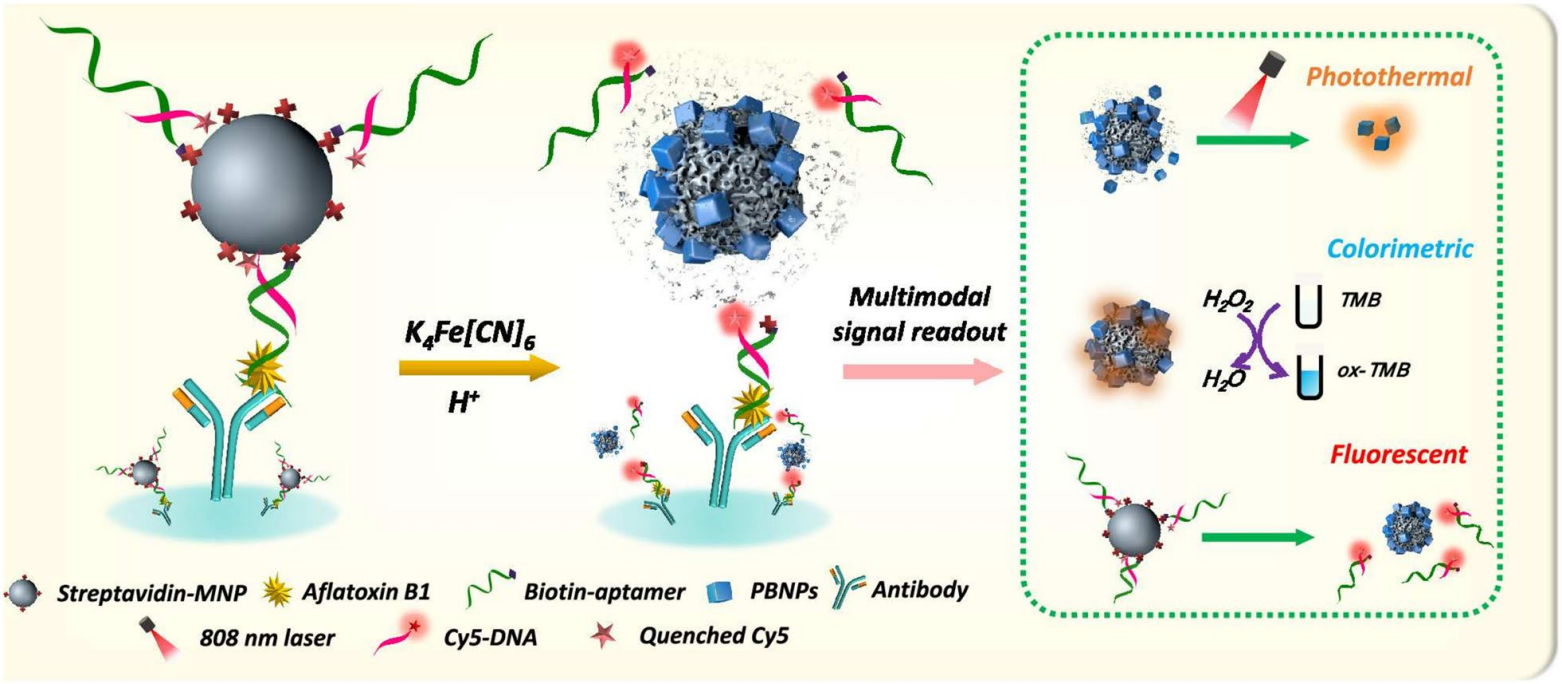
Schematic diagram of multimode detecting AFB1. Reprinted with permission from [90]

The single signal mode is prone to false-negative/positive results caused by differences in operating conditions and environment. In contrast, multi-mode detection can offset interferences, reduce false results through self-correction, and yield more precise outcomes [90, 100]. Huang et al. [101] developed a colorimetric/photothermal dual-mode immunoassay method based on Pt supported on nitrogen-doped carbon (Pt-CN) for monitoring AFB1 in peanut samples. After competitive immunoreactivity of glucose oxidase (GOx)-labeled antigen with AFB1, the GOx loaded on the well plates can catalyze the formation of H_2_O_2_ from glucose. Subsequently, the Pt-CN with POD-like activity can oxidize TMB to blue ox-TMB, producing a colorimetric signal. On the other hand, the ox-TMB, as a photothermal agent, can convert light to heat under near-infrared (NIR) irradiation, generating a photothermal signal. The LOD values are 0.22 pg/mL and 0.76 pg/mL for the colorimetric and photothermal assays, respectively. The fluorescence method is regarded as one of the most sensitive of the optical methods. Lu et al. [90] designed a photothermal/colorimetric/fluorescent multimodal NLISA to portably and ultra-sensitively detect AFB1. As shown in Fig. 11, magnetic nanoparticles (MNPs) combined with aptamers were immobilized on the well plates via antibody and AFB1. In the presence of K_4_[Fe(CN)_6_] and HCl, MNPs as precursors can form Prussian blue nanoparticles (PBNPs) that possessed both excellent POD-like activity and photothermal effect. Particularly, MNPs also acted as the quencher to decrease the fluorescence of the dye (Cy5), which was restored upon the formation of PBNPs, enabling fluorescence detection of FAB1, with an extremely low LOD of 0.54 fg/mL. The established strategy was feasible for the qualitative and quantitative determination of AFB1 in the actual samples on the spot.

#### Ochratoxin A

**Fig. 12:**
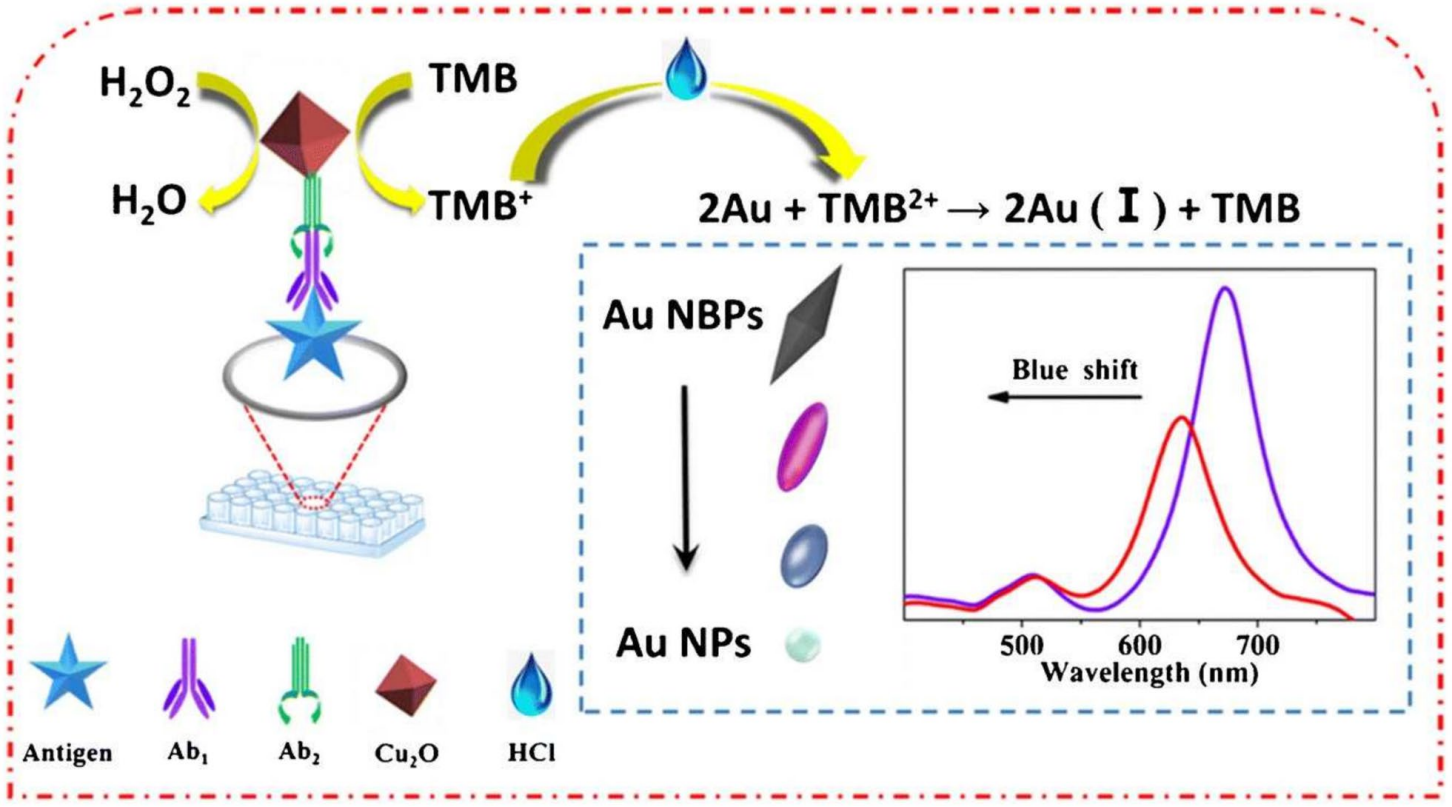
Schematic diagram of multi-colorimetric detecting OTA. Reprinted with permission from [110]

Ochratoxins are a series of mycotoxins generated by *Penicillium* and *Aspergillus*. Among them, ochratoxin A (OTA) is the most poisonous to human health. It possesses various toxicity in animals and humans, including nephrotoxicity, hepatotoxicity, immunotoxicity, teratogenicity, and carcinogenicity [100, 102, 103]. Recently, studies have shown that OTA is also a potent neurotoxin that is considered to be a causative agent of neurodegenerative diseases, and the brain is one of the main target organs of its damage [104]. As mentioned, dual-mode analysis has the advantage of better sensitivity and more accurate results, which was employed in most of the nanozymes-based detection methods of OTA [100, 105–109]. Li et al. [109] developed a SERS/colorimetric dual-mode method for the detection of OTA using Pd–Pt bimetallic nanocrystals (Pd–Pt NRs) conjugated with aptamers as recognition probes. Since the Pd–Pt NRs with POD-like activity can catalyze the oxidation of TMB to ox-TMB that exhibited a strong SERS signal, the SERS and colorimetric detection of OTA can be achieved with LODs of 0.042 nM and 0.097 nM, respectively. The developed method was applied to detect OTA in grape samples, and the results are in consistent with that of UPLC-MS/MS analysis. Zheng et al. [100] established a colorimetric/fluorescence immunoassay method for detecting OTA based on the OXD-like activity of cerium-based nanoparticles (CPNs(IV)) and the fluorescence properties of CPNs(III). The LODs of colorimetric and fluorescence methods are 0.962 ng/mL and 0.404 ng/mL, with recoveries in corn samples ranging from 99.12–102.60% to 97.60–103.55%, respectively. In addition, to improve the accuracy of visual judgments, colorimetric immunoassays with multiple color changes have also been reported. Gold nanomaterials have garnered attention for the color of their solutions, which largely depends on their shape and size [105, 107, 110]. Zhu et al. [110] synthesized octahedral Cu_2_O nanoparticles with POD-like activity, which can oxidize TMB to TMB^2+^ in the presence of H_2_O_2_ and HCl. Based on the significant color change resulted from the etching of TMB^2+^ on gold nano bipyramids (Au NBPs), a multi-colorimetric immunoassay was developed to monitoring of OTA in millet samples with a LOD of 0.47 ng/L (Fig. 12).

#### Zearalenone

Zearalenone (ZEN) is a kind of mycotoxin with estrogenic activity, which can compete with the natural estrogen, resulting in the reproductive dysfunction of animals [83]. In addition, ZEN may cause other toxic effects involving hepatotoxicity, immunotoxicity, genotoxicity, and carcinogenicity [83, 111–114111‒114]. Sun et al. [114] developed a colorimetric method for the detection of ZEN based on the inhibition of ZEN aptamer on the POD-like activity of AuNPs. After the addition of ZEN, the aptamer bound to it preferentially with the restoration of AuNPs activity. The LOD value of the method is 10 ng/mL and the recovery in spiked corn is in the range of 92%‒102%. Bimetallic nanoparticles are superior to monometallic nanoparticles in terms of catalytic activity [108, 109]. Liu et al. [113] synthesized encapsulated AuPt nanoparticles hydrogel by ZEN aptamer and complementary DNA as crosslinkers. In the presence of ZEN, it will preferentially combine with the aptamer, destroying the hydrogel structure and then releasing the AuPt nanozymes to complete the catalytic reaction. Therefore, a highly sensitive colorimetric method for the determination of ZEN was established with a LOD of 0.6979 ng/mL, which was applied to detect ZEN in corn and wheat samples. However, metal nanoparticles alone are prone to aggregation, resulting in reduced catalytic sites and lower catalytic activity. Anchoring it to a carrier material is an effective solution to this problem [91]. For example, utilizing Ti_3_C_2_T_x_ nanosheet as a carrier material, Huang et al. [112] prepared a Ti_3_C_2_T_x_/AuNPs nanocomposite with enhanced POD-like activity, which can be used as an immunoprobe to detect ZEN. Employing Ti_3_C_2_T_x_/AuNP to catalyze the oxidation of TMB and the strong NIR-driven photothermal effect of ox-TMB, the immunoassay achieved ultrasensitive colorimetric and photothermal dual-mode detection of ZEN with LODs of 0.15 pg/mL and 0.48 pg/mL, respectively. Furthermore, the dual-mode strategy was employed for the analysis of ZEN in three contaminated cereal samples, and the results are in good agreement with UPLC-MS/MS analysis.

### Detection of organophosphorus pesticide

Organophosphorus pesticide exposure primarily causes chronic or acute toxicity in humans, plants, and animals through inhibiting cholinesterase activity and leading to acetylcholine accumulation [115]. The hazards of OPs on human beings are generally dominated by acute toxicity, which is manifested by a series of neurotoxic symptoms like sweating, tremors, confusion, speech disorders, and in severe cases, respiratory paralysis and even death [116]. As summarized in Table 5, the strategies for detecting OPs based on nanozymes are categorized as follows: (1) direct influence of OPs on the activity of nanozymes [117–121]; (2) nanozymes with organophosphorus hydrolase (OPH)-like or phosphatase-like activity hydrolyze OPs into products that produce signals [122–131]; (3) nanozymes combine with enzymes that can be inhibited by OPs such as acetylcholinesterase (AChE), alkaline phosphatase (ALP), and ACP [132–141]; (4) nanozymes combine with antibodies or aptamers [142, 143]. Since a review of comprehensive and systematic description of detecting OPs has been reported [10], this paper will not delve into too much detail.

**Table 5:** Summary of detection of organophosphorus pesticide in plants based on nanozymes

Nanozyme/activity	Analyte	Method	Sample	LOD(µM)	Linear range(µM)	Refs.
DTAB-ZnTPyP/POD	Trichlorfon/dichlorvos/thimet	Colorimetric	Apple juice, cabbage, human plasma, *Chrysanthemum morifolium*, *Atractylodes macrocephala*, *Lilium brownie*, and soil	0.25/1.02/0.66 μg/L	1–35/5–45/1–40 μg/L	[117]
Pt NPs/Fe-MOF/POD	Dichlorvos	Colorimetric	Apple and tomato	2.9 pg/mL	0.01–10.0 ng/mL	[137]
DPA-Ce-GMP/OXD	Dimethoate	Colorimetric	Leaf lettuce, brassica campestris, and cucumber	0.024 μg/L	0.03–80 μg/L	[138]
FCC/Sm-CeO_2_/OPH	Methyl paraoxon/Ni^2+^	Fluorescence Colorimetric	Ginseng Radix et Rhizoma Rubra, Nelumbinis Semen, and water	1.25/0.01 –	1.25–60/0.1–8 14.3–285/2.85–285	[127]
CeO_2_/OPH	Methyl paraoxon	Electrochemistry	Nelumbinis Semen, *Coix lacryma-jobi*, and *Adenophora stricta*	0.06	0.1–100	[122]
CFP/Sm-CeO_2_/OPH	Methyl paraoxon	Fluorescence	*Poria cocos* and Coicis Semen	1.0	2–50	[130]
CDs/nanoceria/phosphatase	Methyl paraoxon	Fluorescence	*Panax quinquefolius* and water	0.375	1.125–26.25	[125]
Nanoceria/OPH	Methyl paraoxon	Colorimetric/spectroscopic	Nelumbinis Semen, Armeniacae Semen Amarum, and Dioscoreae Rhizoma	0.42	2.1–21/0.42–42	[129]
NiCo_2_O_4_-PAMAM-peptide/PTE	Methyl paraoxon/Ethyl paraoxon	Electrochemistry	*Brassica chinensis*, tomatoes, and broccoli	0.08/0.16	0.2–100/0.5–100	[131]
Au NBPs@Fe-MOF/POD	Ethyl paraoxon	Colorimetric	Apple peel and lake water	0.01 μg/mL	0.01–0.8 μg/mL	[136]
Au-pCeO_2_/phosphatase	Methyl parathion	Colorimetric	Pears and lettuces	0.5	5–200	[123]
ZrO_2_/CeO_2_/PAA/phosphatase	Methyl parathion	Colorimetric	Corn	0.021 × 10^–3^	0.076 × 10^–3^–76 × 10^–3^	[128]
Mn SAN/SOD	Acetamiprid	Chemiluminescence	*Glycyrrhiza uralensis* and *Astragalus membranaceus*	0.3 pg/mL	1.0–10000 pg/mL	[143]
Ni-NPC/POD	Carbaryl	Colorimetric	Pakchoi and rape	1.5 ng/mL	5–100 ng/mL	[156]
g-C_3_N_4_/BiFeO_3_NCs/POD	Chlorpyrifos/carbaryl	Colorimetric Chemiluminescence	*Salvia miltiorrhiza*, *Codonopsis pilosula*, and lake water	– 0.033 ng/mL	– 1.0–60/1.0–40 ng/mL	[142]
Ir(III)/GO/POD	Pirimicarb	Colorimetric	Pakchoi and apple	0.00281	0.01–0.3	[157]
AuNCs@ZIF-8/POD	OPs	Colorimetric Fluorescence	Lettuce extract and water	0.3 μg/L 0.67 μg/L	0.75 μg/L–75 mg/L 0.75 μg/L–100 mg/L	[132]
SA-Fe-NZ/POD	OPs	Colorimetric/ Electrochemistry	Cucumber, spinach, leek, and broccoli	3.55 × 10^–9^	10^–7^–10^4^	[119]
PANI-MnO_2_/OXD	Glyphosate	Colorimetric	Pear, cucumber, soybean, soil and water	0.39	0.50–50	[139]
SA-CoN_3_/OXD	Glyphosate	Colorimetric	Lake water, apple, pear, peach, and grape	0.79	0–10	[140]
β-CD@DNA-CuNCs/POD	Glyphosate	Colorimetric	Lake water, pease, oats, apple, pakchoi, potato, and tea	0.85 ng/mL	0.02–2 μg/mL	[120]
Mn-ZIF-8/POD	Chlorpyrifos	Colorimetric	Water, cucumber, and pork	54 × 10^–6^	0.0001–0.02	[133]
CeO_2_@NC/OPH	Paraoxon	Colorimetric	Garlic chives	–	3.0–100.0	[124]
Cu-C_3_N_4_/POD	Paraoxon	Colorimetric	Scallion	0.013	0.1–33	[134]
Fe-PTs/POD	Paraoxon	Colorimetric	Rice, wheat, and Yangtze River water	0.28 ng/mL	0.5–250 ng/mL	[135]
Co_3_O_4_/rGO/PTE	Paraoxon	Colorimetric	Cabbage and river water	0.8	8–140	[126]
Fe–N/C SAzyme/OXD	Malathion	Colorimetric	Lake water, apple, tomato, cabbage, and spinach	0.42 × 10^–3^	0.0005–0.01	[141]
Ag_2_O/OXD	Dimethoate	Colorimetric	Pepper, green bean, and cabbage	14 μg/L	20–160 μg/L	[118]
Fe_3_O_4_/Cu-MOF/LAC	Thiram	Electrochemistry	Pear, apple, broccoli, cucumber, and river water	0.15 × 10^–3^	0.01–3.00	[145]
Cu-BDC-NH_2_/LAC and POD	Pesticides	Sensor arrays	Chilli, pear, celery, tomato, cherry, and nectarine	–	1–100 μg/mL	[121]

DTAB-ZnTPyP, dodecyl trimethylammonium bromide-tetramethyl zinc (4-pyridinyl) porphyrin; POD, peroxidase; FCC, fluorescent carbon based composite; OPH, organophosphorus hydrolase; Sm-CeO_2_, samarium doped cerium oxide; CeO_2_, cerium oxide; SAN, single-atom nanozymes; SOD, superoxide dismutase; g-C_3_N_4_/BiFeO_3_NCs, graphitic carbon nitride/bismuth ferrite nanocomposites; CFP, cerium based fluorescent polymer; CDs, carbon dots; OPs, organophosphorus pesticides; ZIF-8, zeolitic Imidazolate Framework-8; AuNCs, gold nanoclusters; PANI, polyaniline; Cu-BDC-NH_2_, Cu coordinated 2-aminoterephthalic acid; LAC, laccase; SA-CoN_3_, single-atom three-coordinated Co; OXD, oxidase; Mn-ZIF-8, Mn-doped ZIF-8; CeO_2_@NC, CeO_2_ nanoparticles are embedded in N-doped carbon material; Fe–N/C SAzyme, Fe–N/C single-atom nanozymes; Fe_3_O_4_/Cu-MOF, magnetic nanoparticles encapsulated metal–organic framework; Cu-C_3_N_4_, Cu-modified graphitic carbon nitride nanomaterial; Fe-PTs, Fe-containing phosphotungstates; Au-pCeO_2_, gold nanoparticles modified porous cerium oxide nanorods; Au NBPs@Fe-MOF, ultrathin MIL-101-NH_2_(Fe) shell-coated Au nanobipyramide; Pt NPs/Fe-MOF, platinum nanoparticles loaded MIL-88B-NH_2_; PTE, phosphotriesterase; PAMAM, poly(amidoamine); Ni-NPC, Ni, N-codoped porous carbon; ZrO_2_/CeO_2_/PAA, polyacrylic acid coated ZrO_2_/CeO_2_ nanorods; Ir(III)/GO, Ir(III) loaded graphene oxide nanosheet; Co_3_O_4_/rGO, reduced graphene oxide supported Co_3_O_4_ nanoparticles; GMP, guanosine monophosphate; DPA, 2,6-Pyridinedicarboxylic acid; SA-Fe-NZ, single-atom iron nanozyme; β-CD, β-Cyclodextrin; CuNCs, copper nanoclusters

## Challenges and prospects

Nanozymes can overcome some drawbacks of natural enzymes and exhibit higher catalytic activity. At present, some nanozymes have been designed for the analysis of plant samples, but there are still some limitations. To promote the development of nanozymes and their application in the detection of plant samples, the following challenges and prospects are proposed.

1. The nanozymes currently used for phytochemical detection are mainly nanomaterials with POD-like or OXD-like activity. The similar detection mechanisms (inhibition on the catalytic activity of nanozymes by antioxidant properties) make it challenging to achieve high specificity in detection. Therefore, the design of nanozymes with other catalytic properties or multiple reaction mechanisms to improve the selectivity of nanozymes for the detection of phytochemicals deserves further investigation.
2. The variety of components based on nanozymes detection in real samples is limited. In plant samples, complex compositions may affect the activity of nanozymes, leading to inaccurate results. Accordingly, detecting active ingredients in real samples often requires complex pretreatments. Developing nanozymes with specific adsorption or combination with other techniques (e.g., MIP) shows greater prospects.
3. In the detection of hazardous substances (e.g., heavy metal ions and pesticide residues), most samples are spiked with them rather than detecting their actual contents directly. This may be due to the low level of hazardous substances in the original sample and the lack of sensitivity of the assay. Modification of nanomaterials with small molecules (e.g., fluorescein derivatives and vitamin B6) that possess POD-like activity to enhance their enzyme-like activity or enable multiple enzyme activities are anticipated to improve the sensitivity of the assay.
4. Nanozyme-based methods for the detection of mycotoxins are mostly combined with immune methods or aptamers, making them more complex than direct colorimetric or fluorescent assays. Only one study was found on the direct colorimetric detection of AFB1 in corn and peanut samples, but its sensitivity is relatively low [89]. Hence, it is necessary to develop more sensitive and direct methods for easier analysis.
5. Currently, there is less literature on detecting heavy metal ions in plant samples (especially in CHMs) based on nanozymes. CHMs have been extensively used in disease treatment and healthcare for their unique therapeutic effects [144]. However, they are susceptible to contamination by chemicals in the environment. Therefore, evaluating the safety of CHMs using nanozymes is very important.
6. Although many nanozymes can mimic the catalysis activity of natural enzymes, their specificity is still inadequate and requires further optimization. In addition, some soluble transition metal nanozymes may be highly toxic and contaminate environment by releasing toxic metal ions. Consequently, it is important to develop nanozymes with improved specificity and biocompatibility.

## Conclusions

This paper summarizes the applications of nanomaterials with enzyme-like activity in plant samples analysis from 2015 to the present, including the analysis of phytochemicals, organophosphorus pesticides, heavy metal ions, and mycotoxins. Improving the selectivity is a research priority for the detection of phytochemicals, which may be achieved through multimode detection and molecular imprinting. Furthermore, due to the trace levels of contaminates, it is crucial to improve sensitivity for detecting hazardous substances. There are various methods have been reported to achieve this goal, including the cascade reaction of natural enzymes with nanozymes and the enhancement of nanozymes’ catalytic activity through doping with other elements or material modification. In general, designing nanozymes with more enzyme-like activities and improving the specificity and sensitivity in their applications are the focus of future research.

## Data Availability

All data generated or analyzed during this review is included in published articles.

## Abbreviations

AA: Ascorbic acid
AAP: Ascorbic acid 2-phosphate
AAS: Atomic absorption spectrometry
AChE: Acetylcholinesterase
ACP: Acid phosphatase
AFB1: Aflatoxin B1
ALP: Alkaline phosphatase
Ar-MoO_3_NPs: Argon cold plasma surface modified molybdenum trioxide nanoparticles
As: Arsenic
As(III): Arsenic trivalent
As(V): Arsenic pentavalent
AS-IV: Astragaloside-IV
Au NBPs: Gold nano bipyramids
AuNCs: Gold nanoclusters
AuNPs: Gold nanoparticles
Cd: Cadmium
CDs: Carbon dots
CE: Capillary electrophoresis
CHMs: Chinese herbal medicines
CHNPs: Copper hexacyanoferrate nanoparticles
CL: Chemiluminescent
COFs: Covalent organic frameworks
CoOOH: Cobalt oxyhydroxide
CPNs: Cerium-based nanoparticles
Cr: Chromium
CTAB: Cetyltrimethylammonium bromide
CTF: Covalent triazine framework
Cu: Copper
Cu-Guo NRs: Cu-guanosine nanorods
DCM: Dichloromethane
ELISA: Enzyme-linked immunosorbent assay
ELSD: Evaporative light scattering detection
Fe: Iron
Fe_3_O_4_@MOFs: Fe_3_O_4_ and bimetal-organic framework Zn/Mg
GA: Gallic acid
GC: Gas chromatography
GOx: Glucose oxidase
Hg: Mercury
HHTP: 2, 3, 6, 7, 10, 11-Hexahydroxytriphenylene
HNCs: Hollow nanocages
HPLC: High-performance liquid chromatography
HRP: Horseradish peroxidase
ICP: Inductively coupled plasma
K_3_[Fe(CN)_6_]: Potassium hexacyanoferrate(III)
LAC: Laccase
LFIA: Lateral flow immunoassay
LOD: Limit of detection
Mb: Methanobactin
M-CATs: Metal-catecholates
MIPs: Molecularly imprinted polymers
MnO_2_/GQD: Manganese dioxide/graphene quantum dot
MNPs: Magnetic nanoparticles
MOFs: Metal organic frameworks
MRLs: Maximum residue limits
MS: Mass spectrometry
NCH: N-doped hollow carbon microspheres
NIR: Near-infrared
NLISA: Nanozyme-linked immunosorbent assay
N-Mn_3_O_4_ NSps: Nitrogen-doped Mn_3_O_4_ nanospheres
NPs: Nanoparticles
Pd–Pt NRs: Pd–Pt bimetallic nanocrystals
NSs: Nanosheets
OPD: O-Phenylenediamine
OPs: Organophosphorus pesticides
OTA: Ochratoxin A
OVs: Oxygen vacancies
OXD: Oxidase
ox-TMB: Oxidized TMB
Pb: Lead
PB: Prussian blue
PBNPs: Prussian blue nanoparticles
PCA: Principal components analysis
PDA: Polydopamine
PFOS: Perfluorooctane sulfonate
POD: Peroxidase
porph@MOF: Porphyrin-functionalized metal–organic framework
PPO: Polyphenol oxidase
Pt-CN: Pt supported on nitrogen-doped carbon
rGO: Reduced graphene oxide
ROS: Reactive oxygen species
SACu-C-N: Single-atom Cu-C-N
SAzymes: Single-atom nanozymes
SERS: Surface-enhanced Raman spectroscopy
SrTiO_3_: Strontium titanate
TA: Tannic acid
TAC: Total antioxidant capacity
TMB: 3, 3’, 5, 5’-Tetramethylbenzidine
UPLC-MS/MS: Ultra-performance liquid chromatography-tandem mass spectrometry
ZEN: Zearalenone
Zn-MOF: Zn-based metal–organic framework

## References

[1] Yang CJ, Zhao Y, Jiang S, Sun XM, Wang XT, Wang ZB, et al. A breakthrough in phytochemical profiling: ultra-sensitive surface-enhanced Raman spectroscopy platform for detecting bioactive components in medicinal and edible plants. Microchim Acta. 2024;191:286. 10.1007/s00604-024-06360-x 38652378

[2] Rodríguez-Negrete EV, Morales-González Á, Madrigal-Santillán EO, Sánchez-Reyes K, Álvarez-González I, Madrigal-Bujaidar E, et al. Phytochemicals and their usefulness in the maintenance of health. Plants. 2024;13:523. 38498532 10.3390/plants13040523 PMC10892216

[3] Barbieri R, Coppo E, Marchese A, Daglia M, Sobarzo-Sánchez E, Nabavi SF, Nabavi SM. Phytochemicals for human disease: An update on plant-derived compounds antibacterial activity. Microbiol Res. 2017;196:44–68. 28164790 10.1016/j.micres.2016.12.003

[4] Imam H, Wu H, Luo T, Arshad M, Song JY, Xu DX, et al. Phytochemicals and inflammatory bowel disease: a review. Crit Rev Food Sci Nutr. 2022;60:1321–45. 10.1080/10408398.2019.1570913 30729797

[5] Chen X, Yang Z, Xu Y, Liu Z, Liu YF, Dai YT, et al. Progress and prediction of multicomponent quantification in complex systems with practical LC-UV methods. J Pharm Anal. 2023;13:142–55. 36908853 10.1016/j.jpha.2022.11.011 PMC9999300

[6] Gotti R. Capillary electrophoresis of phytochemical substances in herbal drugs and medicinal plants. J Pharm Biomed Anal. 2011;55:775–801. 21183304 10.1016/j.jpba.2010.11.041

[7] Pan ZW, Gong TY, Liang P. Heavy metal exposure and cardiovascular disease. Circ Res. 2024;134:1160–78. 38662861 10.1161/CIRCRESAHA.123.323617

[8] Si LX, Wu Q, Jin YL, Wang Z. Research progress in the detection of trace heavy metal ions in food samples. Front Chem. 2024;12:1423666. 38867762 10.3389/fchem.2024.1423666 PMC11168114

[9] Chinese Pharmacopoeia Commission. Guiding principles for the formulation of the limit of harmful residues in traditional Chinese medicine 9302. In: Pharmacopoeia of People’s Republic of China Part 4. Beijing: China Medical Science Press; 2020. p. 520–2.

[10] Zhao FN, Wang L, Li MY, Wang M, Liu GY, Ping JF. Nanozyme-based biosensor for organophosphorus pesticide monitoring: functional design, biosensing strategy, and detection application. TrAC Trends Anal Chem. 2023;165: 117152.

[11] Bedair H, Rady HA, Hussien AM, Pandey M, Apollon W, Alkafaas SS, Ghosh S. Pesticide detection in vegetable crops using enzyme inhibition methods: a comprehensive review. Food Anal Methods. 2022;15:1979–2000.

[12] National Health Commission of the People’s Republic of China. National food safety standard-Maximum residue limits for pesticides in food. GB 2763‒2021; 2021.

[13] Zhang XL, Wu D, Zhou XX, Yu YX, Liu JC, Hu N, et al. Recent progress on the construction of nanozymes-based biosensors and their applications to food safety assay. Trends Anal Chem. 2019;121: 115668.

[14] Agriopoulou S, Stamatelopoulou E, Varzakas T. Advances in analysis and detection of major mycotoxins in foods. Foods. 2020;9:518. 32326063 10.3390/foods9040518 PMC7230321

[15] Wei H, Wang EK. Nanomaterials with enzyme-like characteristics (nanozymes): next-generation artificial enzymes. Chem Soc Rev. 2013;42:6060–93. 23740388 10.1039/c3cs35486e

[16] Wang ZR, Zhang RF, Yan XY, Fan KL. Structure and activity of nanozymes: inspirations for de novo design of nanozymes. Mater Today. 2020;41:81–119.

[17] Huang X, Zhang ST, Tang YJ, Zhang XY, Bai Y, Pang H. Advances in metal-organic framework-based nanozymes and their applications. Coord Chem Rev. 2021;449: 214216.

[18] Yang WP, Yang X, Zhu LJ, Chu HS, Li XY, Xu WT. Nanozymes: Activity origin, catalytic mechanism, and biological application. Coord Chem Rev. 2021;448: 214170.

[19] Zhang DH, Kukkar D, Kaur H, Kim KH. Recent advances in the synthesis and applications of single-atom nanozymes in food safety monitoring. Adv Colloid Interface Sci. 2023;319: 102968. 37582302 10.1016/j.cis.2023.102968

[20] Wang KD, Meng XQ, Yan XY, Fan KL. Nanozyme-based point-of-care testing: revolutionizing environmental pollutant detection with high efficiency and low cost. Nano Today. 2024;54: 102145.

[21] Ling ZZ, Yang JY, Zhang YY, Zeng DP, Wang Y, Tian YX, et al. Applications of advanced materials in the pretreatment and rapid detection of small molecules in foods: a review. Trends Food Sci Technol. 2023;141: 104175.

[22] Chen HY, Zhang L, Hu Y, Zhou CS, Lan W, Fu HY, She YB. Nanomaterials as optical sensors for application in rapid detection of food contaminants, quality and authenticity. Sens Actuators B Chem. 2021;329: 129135.

[23] Wang JL, Chai TQ, Chen LX, Chen GY, Chen H, Yang FQ. Manganese coordination polymer nanoparticles with excellent oxidase-like activity for the rapidly and selectively colorimetric detection of glutathione. Microchem J. 2024;199: 110207.

[24] Xu X, Ma MY, Zhou XY, Zhao X, Feng DM, Zhang L. Portable hydrogel kits made with bimetallic nanozymes for point-of-care testing of perfluorooctanesulfonate. ACS Appl Mater Interfaces. 2024;16:15959–69. 38511635 10.1021/acsami.4c00844

[25] Yang Y. Phytochemicals and health. In: Zhang L, editor. Nutritional toxicology. Springer Nature Singapore: Singapore; 2022. p. 309–54.

[26] Kumar A, Nirmal P, Kumar M, Jose A, Tomer V, Emel OZ, et al. Major phytochemicals: recent advances in health benefits and extraction method. Molecules. 2023;28:887. 36677944 10.3390/molecules28020887 PMC9862941

[27] Sharma BR, Kumar V, Gat Y, Kumar N, Parashar A, Pinakin DJ. Microbial maceration: a sustainable approach for phytochemical extraction. 3 Biotech. 2018;8:401. 30221114 10.1007/s13205-018-1423-8 PMC6128812

[28] Zhou T, Chen DQ, Li HR, Ge DH, Chen XJ. Enhanced oxidase mimic activity of raspberry-like N-doped Mn_3_O_4_ with oxygen vacancies for efficient colorimetric detection of gallic acid coupled with smartphone. Food Chem. 2024;447: 138919. 38452538 10.1016/j.foodchem.2024.138919

[29] Chen LY, Yang J, Chen W, Sun SG, Tang H, Li YC. Perovskite mesoporous LaFeO_3_ with peroxidase-like activity for colorimetric detection of gallic acid. Sens Actuators B Chem. 2020;321: 128642.

[30] Xie XY, Chen XF, Wang YH, Zhang MS, Fan YX, Yang XP. High-loading Cu single-atom nanozymes supported by carbon nitride with peroxidase-like activity for the colorimetric detection of tannic acid. Talanta. 2023;257: 124387. 36841014 10.1016/j.talanta.2023.124387

[31] Wu CH, Qin ZY, Liu YX, Qin XG, Liu G, Wei XL, Zhang HZ. Amorphous iron-catecholates featuring efficient peroxidase-like activity for quick colorimetric detection of tannic acid. LWT. 2024;197: 115896.

[32] Liu YG, Ye HL, Ying MH, Lin X, Jia X, Pan HB. In-situ growth of SrTiO_3_ nanosheets on graphene oxide for colorimetric detection of tannins in tea and behavior of active oxygen radicals in the nanozymatic process. Colloids Surf A. 2023;675: 132109.

[33] Zhang JK, Yang Y, Qin FM, Hu TT, Zhao XS, Zhao SC, et al. Catalyzing generation and stabilization of oxygen vacancies on CeO_2−x_ nanorods by Pt nanoclusters as nanozymes for catalytic therapy. Adv Healthcare Mater. 2023;12:2302056. 10.1002/adhm.202302056 PMC11468536 37708844

[34] Chen LL, Song JQ, Wang L, Li XT, Hao X, Zhang HP, Fan TJ. Fabrication of a dual mimetic enzyme sensor based on gold nanoparticles modified with Cu(II)-coordinated methanobactin for gallic acid detection. J Food Meas Charact. 2024;18:3142–59.

[35] Wu SY, Zhang P, Jiang ZW, Zhang WD, Gong X, Wang Y. Enhanced peroxidase-like activity of CuS hollow nanocages by plasmon-induced hot carriers and photothermal effect for the dual-mode detection of tannic acid. ACS Appl Mater Interfaces. 2022;14:40191–9. 36004449 10.1021/acsami.2c08698

[36] He J, Yang L, Zhang Y, Li RH, Wu JJ, Cao QQ, et al. Pd-Pt-Ru nanozyme with peroxidase-like activity for the detection of total antioxidant capacity. Anal Methods. 2022;15:8–16. 36484272 10.1039/d2ay01560a

[37] Zhang JJ, Li YF, Gong X, Wang Y, Fu WS. Colorimetric detection of total antioxidants in green tea with oxidase-mimetic CoOOH nanorings. Colloids Surf B. 2022;218: 112711. 10.1016/j.colsurfb.2022.112711 35907355

[38] Facure MHM, Andre RS, Mercante LA, Correa DS. Colorimetric detection of antioxidants in food samples using MnO_2_/graphene quantum dot composites with oxidase-like activity. ACS Appl Nano Mater. 2022;5:15211–9.

[39] Davoodi-Rad K, Shokrollahi A, Shahdost-Fard F, Azadkish K, Madani-Nejad E. A smartphone-based colorimetric assay using Cu-tannic acid nanosheets (Cu-TA NShs) as a laccase-mimicking nanozyme for visual detection of quercetin in vegetables. Microchim Acta. 2024;191:168. 10.1007/s00604-024-06238-y 38418635

[40] Mahmoudi S, Chaichi MJ, Shamsipur M, Nazari OL, Samadi Maybodi AR. Modification of bimetal Zn/Mg MOF with nanoparticles Fe_3_O_4_ and Fe_3_O_4_@SiO_2_, investigation of the peroxidase-like activity of these compounds by calorimetry and fluorimetry methods. Heliyon. 2023;9: e12866. 36718154 10.1016/j.heliyon.2023.e12866 PMC9883189

[41] Ye KX, Xu SF, Zhou QQ, Wang ST, Xu ZG, Liu ZM. Advances in molecular imprinting technology for the extraction and detection of quercetin in plants. Polymers. 2023;15:2107. 37177253 10.3390/polym15092107 PMC10180927

[42] Cao XY, Zhao S, Liu XW, Zhu XX, Gao Y, Liu QY. CeO_2_/Co_3_O_4_@N-doped hollow carbon microspheres with improved peroxidase-like activity for the determination of quercetin. Anal Bioanal Chem. 2022;414:4767–75. 35524002 10.1007/s00216-022-04100-9

[43] Wang JH, Huang RL, Qi W, Su RX, He ZM. Construction of biomimetic nanozyme with high laccase- and catecholase-like activity for oxidation and detection of phenolic compounds. J Hazard Mater. 2022;429: 128404. 35236027 10.1016/j.jhazmat.2022.128404

[44] Rashtbari S, Dehghan G, Amini M, Khorram S, Khataee A. A sensitive colori/fluorimetric nanoprobe for detection of polyphenols using peroxidase-mimic plasma-modified MoO_3_ nanoparticles. Chemosphere. 2022;295: 133747. 35120949 10.1016/j.chemosphere.2022.133747

[45] Feng GJ, Yang Y, Zeng JT, Zhu J, Liu JJ, Wu L, et al. Highly sensitive electrochemical determination of rutin based on the synergistic effect of 3D porous carbon and cobalt tungstate nanosheets. J Pharm Anal. 2022;12:453–9. 35811621 10.1016/j.jpha.2021.09.007 PMC9257437

[46] Davoodi-Rad K, Shokrollahi A, Shahdost-Fard F, Azadkish K. Copper-guanosine nanorods (Cu-Guo NRs) as a laccase mimicking nanozyme for colorimetric detection of rutin. Biosensors. 2023;13:374. 36979586 10.3390/bios13030374 PMC10046739

[47] Tan HN, Zhao YX, Xu XT, Sun Y, Li YH, Du JX. A covalent triazine framework as an oxidase mimetic in the luminol chemiluminescence system: application to the determination of the antioxidant rutin. Microchim Acta. 2019;187:42. 10.1007/s00604-019-4058-5 31832861

[48] Mahmoudi S, Chaichi MJ, Shamsipur M, Nazari OL, Samadi-Maybodi A. Fe_3_O_4_ and bimetal-organic framework Zn/Mg composite peroxide-like catalyze luminol chemiluminescence for specific measurement of atropine in Datura plant. Luminescence. 2023;38:1711–9. 37455562 10.1002/bio.4557

[49] Belbruno JJ. Molecularly imprinted polymers. Chem Rev. 2018;119:94–119. 30246529 10.1021/acs.chemrev.8b00171

[50] Zhang ZJ, Li YQ, Zhang XH, Liu JW. Molecularly imprinted nanozymes with faster catalytic activity and better specificity†. Nanoscale. 2019. 10.1039/C8NR09816F. 30820498 10.1039/c8nr09816f

[51] Chen GY, Chen LX, Gao J, Chen CY, Guan JL, Cao ZM, et al. A novel molecularly imprinted sensor based on CuO nanoparticles with peroxidase-like activity for the selective determination of astragaloside-IV. Biosensors. 2023;13:959. 37998134 10.3390/bios13110959 PMC10669883

[52] Chai XY, Gu YQ, Lv L, Chen C, Feng F, Cao Y, et al. Screening of immune cell activators from Astragali Radix using a comprehensive two-dimensional NK-92MI cell membrane chromatography/C18 column/time-of-flight mass spectrometry system. J Pharm Anal. 2022;12:725–32. 36320599 10.1016/j.jpha.2022.05.006 PMC9615523

[53] Kwon HJ, Park YD. Determination of astragalin and astragaloside content in Radix Astragali using high-performance liquid chromatography coupled with pulsed amperometric detection. J Chromatogr A. 2012;1232:212–7. 22209546 10.1016/j.chroma.2011.12.035

[54] Li JD, Li P, Wu D, Wang ZX, Liu Y, Wang XC, Zhou JW. Effects of different processing methods on the content of astragaloside in danggui buxue decoction detection by high performance liquid chromatography-evaporative light scattering detection. Mater Express. 2022;12:1004–11.

[55] Yang MH, Zhang M, Jia MY. Optical sensor arrays for the detection and discrimination of natural products. Nat Prod Rep. 2023;40:628–45. 36597853 10.1039/d2np00065b

[56] Yuan XH, Cheng SC, Chen LY, Cheng ZY, Liu J, Zhang H, et al. Iron oxides based nanozyme sensor arrays for the detection of active substances in licorice. Talanta. 2023;258: 124407. 36871515 10.1016/j.talanta.2023.124407

[57] Zhang L, Bi XY, Liu XH, He Y, Li LB, You TY. Advances in the application of metal-organic framework nanozymes in colorimetric sensing of heavy metal ions. Nanoscale. 2023;15:12853–67. 37490007 10.1039/d3nr02024j

[58] Xu XC, Luo ZJ, Ye K, Zou XB, Niu XH, Pan JM. One-pot construction of acid phosphatase and hemin loaded multifunctional metal-organic framework nanosheets for ratiometric fluorescent arsenate sensing. J Hazard Mater. 2020;412: 124407. 33548790 10.1016/j.jhazmat.2020.124407

[59] Wang JJ, Tao H, Lu TT, Wu YG. Adsorption enhanced the oxidase-mimicking catalytic activity of octahedral-shape Mn_3_O_4_ nanoparticles as a novel colorimetric chemosensor for ultrasensitive and selective detection of arsenic. J Colloid Interface Sci. 2020;584:114–24. 33069011 10.1016/j.jcis.2020.09.107

[60] Wang LJ, Yang JL, Yan Y, Zhang YS, Xu XC. A smartphone-integrated colorimetric quantitative analysis platform based on oxidase-like Ce(IV)-ATP-Tris CPNs/CNF test strip for detection of inorganic arsenic in rice. Anal Chim Acta. 2022;1227: 340308. 36089319 10.1016/j.aca.2022.340308

[61] Saifullah, Dahlawi S, Naeem A, Iqbal M, Farooq MA, Bibi S, Rengel Z. Opportunities and challenges in the use of mineral nutrition for minimizing arsenic toxicity and accumulation in rice: A critical review. Chemosphere. 2018;194:171‒88. 10.1016/j.chemosphere.2017.11.149 29202269

[62] Zulfiqar U, Farooq M, Hussain S, Maqsood M, Hussain M, Ishfaq M, et al. Lead toxicity in plants: impacts and remediation. J Environ Manage. 2019;250: 109557. 31545179 10.1016/j.jenvman.2019.109557

[63] Li XX, Lan X, Liu W, Cui XW, Cui ZJ. Toxicity, migration and transformation characteristics of lead in soil-plant system: effect of lead species. J Hazard Mater. 2020;395: 122676. 32325342 10.1016/j.jhazmat.2020.122676

[64] Cui YF, Li QL, Yang DZ, Yang YL. Colorimetric-SERS dual-mode sensing of Pb(II) ions in traditional Chinese medicine samples based on carbon dots-capped gold nanoparticles as nanozyme. Spectrochim Acta A Mol Biomol Spectrosc. 2024;313: 124100. 38484642 10.1016/j.saa.2024.124100

[65] Zhang XN, Huang XY, Xu YW, Wang X, Guo ZM, Huang XW, et al. Single-step electrochemical sensing of ppt-level lead in leaf vegetables based on peroxidase-mimicking metal-organic framework. Biosens Bioelectron. 2020;168: 112544. 32892116 10.1016/j.bios.2020.112544

[66] Tang Y, Hu Y, Yang YX, Liu BY, Wu YG. A facile colorimetric sensor for ultrasensitive and selective detection of lead(II) in environmental and biological samples based on intrinsic peroxidase-mimic activity of WS_2_ nanosheets. Anal Chim Acta. 2020;1106:115–25. 32145839 10.1016/j.aca.2020.01.043

[67] Yu Y, Zhang Y, Li WH, Wang ZW, Zhang J. DNA nanocage confined DNAzyme for detection of lead ions coupled with CRISPR-Cas12a system. Chem Eng J. 2024;480: 148177.

[68] Dasary SSR, Jones YK, Barnes SL, Ray PC, Singh AK. Alizarin dye based ultrasensitive plasmonic SERS probe for trace level cadmium detection in drinking water. Sens Actuators B Chem. 2016;224:65–72. 26770012 10.1016/j.snb.2015.10.003 PMC4707966

[69] Li QL, Han QQ, Yang DZ, Li KX, Wang YJ, Chen D, et al. Methylmercury-sensitized “turn on” SERS-active peroxidase-like activity of carbon dots/Au NPs nanozyme for selective detection of ochratoxin A in coffee. Food Chem. 2024;434: 137440. 37725842 10.1016/j.foodchem.2023.137440

[70] Yaseen T, Pu HB, Sun DW. Fabrication of silver-coated gold nanoparticles to simultaneously detect multi-class insecticide residues in peach with SERS technique. Talanta. 2019;196:537–45. 30683402 10.1016/j.talanta.2018.12.030

[71] León Anchustegui VA, Zhu JH, He LY, Bi Y, Dong YY, Liu JH, Wang SH. Coencapsulation of carbon dots and gold nanoparticles over escherichia coli for bacterium assay by surface-enhanced Raman scattering. ACS Appl Bio Mater. 2021;4:597–604.

[72] Li H, Jiang CN, He X, Li CN, Jiang ZL. Aptamer SERS and RRS determination of trace lead ions using nitrogen-doped carbon dot to catalyze the new nano-gold reaction. Spectrochim Acta A Mol Biomol Spectrosc. 2023;303: 123146. 37523850 10.1016/j.saa.2023.123146

[73] Wang HL, Zhang ZH, Chen CQ, Liang AH, Jiang ZL. Fullerene carbon dot catalytic amplification-aptamer assay platform for ultratrace As^3+^ utilizing SERS/RRS/Abs trifunctional Au nanoprobes. J Hazard Mater. 2021;403: 123633. 32827860 10.1016/j.jhazmat.2020.123633

[74] Budnik LT, Casteleyn L. Mercury pollution in modern times and its socio-medical consequences. Sci Total Environ. 2019;654:720–34. 30448663 10.1016/j.scitotenv.2018.10.408

[75] Ge J, Yuan YT, Yang H, Deng RJ, Li ZH, Yang Y. Smartphone-assisted colorimetric sensor based on single-atom Cu-C-N nanozyme for mercury (II) ions detection. Mater Today Chem. 2024;37: 102037.

[76] Song GC, Zhang Q, Liang S, Yao Y, Feng ML, Majid ZNB, et al. Oxidation activity modulation of a single atom Ce-N-C nanozyme enabling a time-resolved sensor to detect Fe^3+^ and Cr^6+^. J Mater Chem C. 2022;10:15656–63.

[77] Guo Q, Huang XR, Huang YJ, Zhang ZW, Li PW, Yu L. Fe-N-C single-atom nanozyme-linked immunosorbent assay for quantitative detection of aflatoxin B1. J Food Compos Anal. 2024;125: 105795.

[78] Zhang Y, Yuan X, Guo XY, Xu H, Zhang DX, Wu ZY, Zhang J. All-in-one zinc-doped Prussian blue nanozyme for efficient capture, separation, and detection of copper Ion (Cu^2+^) in complicated matrixes. Small. 2024;20:2306961. 10.1002/smll.202306961 37803466

[79] Liu Y, Ding D, Zhen YL, Guo R. Amino acid-mediated ‘turn-off/turn-on’ nanozyme activity of gold nanoclusters for sensitive and selective detection of copper ions and histidine. Biosens Bioelectron. 2017;92:140–6. 28213326 10.1016/j.bios.2017.01.036

[80] Xiong YH, Su LJ, He XC, Duan ZH, Zhang Z, Chen ZL, et al. Colorimetric determination of copper ions based on regulation of the enzyme-mimicking activity of covalent triazine frameworks. Sens Actuators B Chem. 2017;253:384–91.

[81] Lee S, Barin G, Ackerman CM, Muchenditsi A, Xu J, Reimer JA, et al. Copper capture in a thioether-functionalized porous polymer applied to the detection of Wilson’s disease. J Am Chem Soc. 2016;138:7603–9. 27285482 10.1021/jacs.6b02515 PMC5555401

[82] Li YY, Mu ZD, Yuan YH, Zhou J, Bai LJ, Qing M. An enzymatic activity regulation-based clusterzyme sensor array for high-throughput identification of heavy metal ions. J Hazard Mater. 2023;454: 131501. 37119573 10.1016/j.jhazmat.2023.131501

[83] Jing SY, Liu CM, Zheng J, Dong ZJ, Guo N. Toxicity of zearalenone and its nutritional intervention by natural products. Food Funct. 2022;13:10374–400. 36165278 10.1039/d2fo01545e

[84] Su ZH, Du T, Liang XF, Wang XZ, Zhao LF, Sun J, et al. Nanozymes for foodborne microbial contaminants detection: mechanisms, recent advances, and challenges. Food Control. 2022;141: 109165.

[85] Xing KY, Shan S, Liu DF, Lai WH. Recent advances of lateral flow immunoassay for mycotoxins detection. TrAC Trends Anal Chem. 2020;133: 116087.

[86] Chinese Pharmacopoeia Commission. Medicinal materials and cut crude drugs In: Pharmacopoeia of People’s Republic of China Part 1. Beijing: China Medical Science Press; 2020.

[87] Lai WQ, Wei QH, Xu MD, Zhuang JY, Tang DP. Enzyme-controlled dissolution of MnO_2_ nanoflakes with enzyme cascade amplification for colorimetric immunoassay. Biosens Bioelectron. 2017;89:645–51. 26725933 10.1016/j.bios.2015.12.035

[88] Marchese S, Polo A, Ariano A, Velotto S, Costantini S, Severino L. Aflatoxin B1 and M1: biological properties and their involvement in cancer development. Toxins. 2018;10:214. 29794965 10.3390/toxins10060214 PMC6024316

[89] Zhang SY, Li H, Xia QH, Yang DZ, Yang YL. Zirconium-porphyrin-MOF-based oxidase-like nanozyme with oxygen vacancy for aflatoxin B1 colorimetric sensing. J Food Sci. 2024;89:3618–28. 38685872 10.1111/1750-3841.17077

[90] Lu D, Jiang H, Zhang GY, Luo Q, Zhao Q, Shi XB. An in situ generated Prussian blue nanoparticle-mediated multimode nanozyme-linked immunosorbent assay for the detection of aflatoxin B1. ACS Appl Mater Interfaces. 2021;13:25738–47. 34043909 10.1021/acsami.1c04751

[91] Zhao YK, Wang XF, Pan SX, Hong F, Lu P, Hu XB, et al. Bimetallic nanozyme-bioenzyme hybrid material-mediated ultrasensitive and automatic immunoassay for the detection of aflatoxin B1 in food. Biosens Bioelectron. 2024;248: 115992. 38184942 10.1016/j.bios.2023.115992

[92] Rushing BR, Selim MI. Aflatoxin B1: a review on metabolism, toxicity, occurrence in food, occupational exposure, and detoxification methods. Food Chem Toxicol. 2019;124:81–100. 30468841 10.1016/j.fct.2018.11.047

[93] Chen PF, Li SL, Jiang CY, Wang ZP, Ma XY. A surface-enhanced Raman scattering aptasensor for output-signal detection of aflatoxin B1 based on peroxidase-like Cu_2_O@Au hybrid nanozyme. Food Biosci. 2023;54: 102885.

[94] Zhou SY, Xu LG, Kuang H, Xiao J, Xu CL. Immunoassays for rapid mycotoxin detection: state of the art. Analyst. 2020;145:7088–102. 32990695 10.1039/d0an01408g

[95] Cai XF, Liang MJ, Ma F, Zhang ZW, Tang XQ, Jiang J, et al. Nanozyme-strip based on MnO_2_ nanosheets as a catalytic label for multi-scale detection of aflatoxin B1 with an ultrabroad working range. Food Chem. 2022;377: 131965. 34979398 10.1016/j.foodchem.2021.131965

[96] Liang MJ, Cai XF, Gao YY, Yan HL, Fu JY, Tang XQ, et al. A versatile nanozyme integrated colorimetric and photothermal lateral flow immunoassay for highly sensitive and reliable *Aspergillus flavus* detection. Biosens Bioelectron. 2022;213: 114435. 35679645 10.1016/j.bios.2022.114435

[97] Zhu X, Tang J, Ouyang XL, Liao Y, Feng HP, Yu JF, et al. A versatile CuCo@PDA nanozyme-based aptamer-mediated lateral flow assay for highly sensitive, on-site and dual-readout detection of aflatoxin B1. J Hazard Mater. 2024;465: 133178. 38064951 10.1016/j.jhazmat.2023.133178

[98] Zhao Q, Lu D, Zhang GY, Zhang D, Shi XB. Recent improvements in enzyme-linked immunosorbent assays based on nanomaterials. Talanta. 2021;223: 121722. 33303168 10.1016/j.talanta.2020.121722

[99] Lai WQ, Guo JQ, Wang YQ, Lin YX, Ye SA, Zhuang JY, Tang DP. Enzyme-controllable just-in-time production system of copper hexacyanoferrate nanoparticles with oxidase-mimicking activity for highly sensitive colorimetric immunoassay. Talanta. 2022;247: 123546. 35594834 10.1016/j.talanta.2022.123546

[100] Zheng XL, Sun LL, Zhao YN, Yang HL, Zhu YH, Zhang JX, et al. A fluorescence and colorimetric dual-mode immunoassay for detection of ochratoxin A based on cerium nanoparticles. Microchem J. 2024;201: 110419.

[101] Huang SY, Lai WQ, Liu BQ, Xu MD, Zhuang JY, Tang DP, Lin YX. Colorimetric and photothermal dual-mode immunoassay of aflatoxin B1 based on peroxidase-like activity of Pt supported on nitrogen-doped carbon. Spectrochim Acta A Mol Biomol Spectrosc. 2023;284: 121782. 36049298 10.1016/j.saa.2022.121782

[102] Zhu HS, Quan Z, Hou HY, Cai Y, Liu WP, Liu YJ. A colorimetric immunoassay based on cobalt hydroxide nanocages as oxidase mimics for detection of ochratoxin A. Anal Chim Acta. 2020;1132:101–9. 32980100 10.1016/j.aca.2020.07.068

[103] Tang JD, Tian B, Tao XQ. A colorimetric aptasensor for detecting ochratoxin A based on label-free aptamer and gold nanozyme. Anal Sci. 2023;39:1623–6. 37566171 10.1007/s44211-023-00404-7

[104] Obafemi BA, Adedara IA, Rocha JBT. Neurotoxicity of ochratoxin A: molecular mechanisms and neurotherapeutic strategies. Toxicology. 2023;497–498: 153630. 37709162 10.1016/j.tox.2023.153630

[105] Chen MT, Huang XM, Chen YX, Cao YR, Zhang SS, Lei HT, et al. Shape-specific MOF-derived Cu@Fe-NC with morphology-driven catalytic activity: Mimicking peroxidase for the fluorescent- colorimetric immunosignage of ochratoxin. J Hazard Mater. 2023;443: 130233. 36308933 10.1016/j.jhazmat.2022.130233

[106] Chen MT, Liu ZX, Guan YY, Chen YX, Liu WP, Liu YJ. Zeolitic imidazolate frameworks-derived hollow Co/N-doped CNTs as oxidase-mimic for colorimetric-fluorescence immunoassay of ochratoxin A. Sens Actuators B Chem. 2022;359: 131609.

[107] Zhu HS, Cai Y, Qileng A, Quan Z, Zeng W, He KY, Liu YJ. Template-assisted Cu_2_O@Fe(OH)_3_ yolk-shell nanocages as biomimetic peroxidase: a multi-colorimetry and ratiometric fluorescence separated-type immunosensor for the detection of ochratoxin A. J Hazard Mater. 2021;411: 125090. 33453667 10.1016/j.jhazmat.2021.125090

[108] Ke CX, Wu Y, Song ZC, Zheng ME, Zhu HD, Guo HL, et al. A novel competitive fluorescence colorimetric dual-mode immunosensor for detecting ochratoxin A based on the synergistically enhanced peroxidase-like activity of AuAg NCs-SPCN nanocomposite. Food Chem. 2024;437: 137930. 37944394 10.1016/j.foodchem.2023.137930

[109] Li M, Wang H, Yu XD, Jia XD, Zhu C, Liu JH, et al. A sensitive and simple competitive nanozyme-linked apta-sorbent assay for the dual-mode detection of ochratoxin A. Analyst. 2022;147:2215–22. 35467672 10.1039/d1an02335g

[110] Zhu HS, Liu CH, Liu XX, Quan Z, Liu WP, Liu YJ. A multi-colorimetric immunosensor for visual detection of ochratoxin A by mimetic enzyme etching of gold nanobipyramids. Microchim Acta. 2021;188:62. 10.1007/s00604-020-04699-5 33534035

[111] Qiao WL, He BS, Yang J, Ren WJ, Zhao RY, Zhang YR, et al. Pt@AuNF nanozyme and horseradish peroxidase-based lateral flow immunoassay dual enzymes signal amplification strategy for sensitive detection of zearalenone. Int J Biol Macromol. 2024;254: 127746. 37923041 10.1016/j.ijbiomac.2023.127746

[112] Huang N, Sheng W, Jin Z, Bai DM, Sun MY, Ren LS, et al. Colorimetric and photothermal dual-mode immunosensor based on Ti_3_C_2_T_x_/AuNPs nanocomposite with enhanced peroxidase-like activity for ultrasensitive detection of zearalenone in cereals. Microchim Acta. 2023;190:479. 10.1007/s00604-023-06073-7 37994918

[113] Liu QW, Zhou LL, Xin SY, Yang QL, Wu W, Hou XD. Poly (ionic liquid) cross-linked hydrogel encapsulated with AuPt nanozymes for the smartphone-based colorimetric detection of zearalenone. Food Chem X. 2024;22: 101471. 38846799 10.1016/j.fochx.2024.101471 PMC11154200

[114] Sun SM, Zhao R, Feng SM, Xie YL. Colorimetric zearalenone assay based on the use of an aptamer and of gold nanoparticles with peroxidase-like activity. Microchim Acta. 2018;185:535. 10.1007/s00604-018-3078-x 30406298

[115] Sidhu GK, Singh S, Kumar V, Dhanjal DS, Datta S, Singh J. Toxicity, monitoring and biodegradation of organophosphate pesticides: a review. Crit Rev Environ Sci Technol. 2019;49:1135–87.

[116] Richardson JR, Fitsanakis V, Westerink RHS, Kanthasamy AG. Neurotoxicity of pesticides. Acta Neuropathol. 2019;138:343–62. 31197504 10.1007/s00401-019-02033-9 PMC6826260

[117] Deng GQ, Chen HY, Shi Q, Ren LX, Liang K, Long WJ, et al. Colorimetric assay based on peroxidase-like activity of dodecyl trimethylammonium bromide-tetramethyl zinc (4-pyridinyl) porphyrin for detection of organophosphorus pesticides. Microchim Acta. 2022;189:375. 10.1007/s00604-022-05430-2 36074197

[118] Zhan XQ, Tang Y, Liu YY, Tao H, Wu YG. A novel colorimetric strategy for rapid detection of dimethoate residue in vegetables based on enhancing oxidase-mimicking catalytic activity of cube-shape Ag_2_O particles. Sens Actuators B Chem. 2022;361: 131720.

[119] Wang GX, Liu J, Dong HW, Geng LJ, Sun JS, Liu JJ, et al. A dual-mode biosensor featuring single-atom Fe nanozyme for multi-pesticide detection in vegetables. Food Chem. 2023;437: 137882. 37948799 10.1016/j.foodchem.2023.137882

[120] Tai SM, Qian ZJ, Ren HX, Barimah AO, Peng CF, Wei XL. Highly selective and sensitive colorimetric detection for glyphosate based on β-CD@DNA-CuNCs enzyme mimics. Anal Chim Acta. 2022;1222: 339992. 35934420 10.1016/j.aca.2022.339992

[121] Song DH, Tian T, Wang L, Zou YT, Zhao LZ, Xiao J, et al. Multi-signal sensor array based on a fluorescent nanozyme for broad-spectrum screening of pesticides. Chem Eng J. 2024;482: 148784.

[122] Sun YZ, Wei JC, Zou J, Cheng ZH, Huang ZM, Gu LQ, et al. Electrochemical detection of methyl-paraoxon based on bifunctional cerium oxide nanozyme with catalytic activity and signal amplification effect. J Pharm Anal. 2020;11:653–60. 34765279 10.1016/j.jpha.2020.09.002 PMC8572677

[123] Zhao FN, Li MY, Wang L, Wang M. A colorimetric sensor enabled with heterogeneous nanozymes with phosphatase-like activity for the residue analysis of methyl parathion. Foods. 2023;12:2980. 37569249 10.3390/foods12152980 PMC10418809

[124] Gai PP, Pu L, Wang C, Zhu DQ, Li F. CeO_2_@NC nanozyme with robust dephosphorylation ability of phosphotriester: a simple colorimetric assay for rapid and selective detection of paraoxon. Biosens Bioelectron. 2022;220: 114841. 36323162 10.1016/j.bios.2022.114841

[125] Wei JC, Yang Y, Dong JY, Wang SP, Li P. Fluorometric determination of pesticides and organophosphates using nanoceria as a phosphatase mimic and an inner filter effect on carbon nanodots. Microchim Acta. 2019;186:66. 10.1007/s00604-018-3175-x 30627852

[126] Wang T, Wang JN, Yang Y, Su P, Yang Y. Co_3_O_4_/reduced graphene oxide nanocomposites as effective phosphotriesterase mimetics for degradation and detection of paraoxon. Ind Eng Chem Res. 2017;56:9762–9.

[127] Luo M, Chen L, Wei JC, Cui XP, Cheng ZH, Wang T, et al. A two-step strategy for simultaneous dual-mode detection of methyl-paraoxon and Ni (II). Ecotoxicol Environ Saf. 2022;239: 113668. 35623151 10.1016/j.ecoenv.2022.113668

[128] Wu XC, Wei JH, Wu CY, Lv GP, Wu LN. ZrO_2_/CeO_2_/polyacrylic acid nanocomposites with alkaline phosphatase-like activity for sensing. Spectrochim Acta A Mol Biomol Spectrosc. 2021;263: 120165. 34304012 10.1016/j.saa.2021.120165

[129] Wei JC, Yang LL, Luo M, Wang YT, Li P. Nanozyme-assisted technique for dual mode detection of organophosphorus pesticide. Ecotoxicol Environ Saf. 2019;179:17–23. 31022651 10.1016/j.ecoenv.2019.04.041

[130] Wei JC, Xue Y, Dong JY, Wang SP, Hu H, Gao H, et al. A new fluorescent technique for pesticide detection by using metal coordination polymer and nanozyme. Chin Med. 2020;15:22. 32175000 10.1186/s13020-020-00304-2 PMC7063803

[131] Yang YY, Hao SJ, Lei XM, Chen JN, Fang GZ, Liu JF, et al. Design of metalloenzyme mimics based on self-assembled peptides for organophosphorus pesticides detection. J Hazard Mater. 2022;428: 128262. 35051771 10.1016/j.jhazmat.2022.128262

[132] Cai Y, Zhu HS, Zhou WC, Qiu ZY, Chen CC, Qileng AR, et al. Capsulation of AuNCs with AIE effect into metal-organic framework for the marriage of a fluorescence and colorimetric biosensor to detect organophosphorus pesticides. Anal Chem. 2021;93:7275–82. 33957044 10.1021/acs.analchem.1c00616

[133] Feng YY, Hu P, Wang M, Sun XB, Pan W, Wang JP. Introducing Mn into ZIF-8 nanozyme for enhancing its catalytic activities and adding specific recognizer for detection of organophosphorus pesticides. Microchim Acta. 2023;190:437. 10.1007/s00604-023-06016-2 37843605

[134] Chang GR, Li SR, Wang YQ, Ran QX, Tan Q, Gou S, et al. Cu-C_3_N_4_ nanoenzyme-based freezing-dried bioactive capsule integrated with 3D-printed smartphone platform for visual detection of organophosphorus pesticides paraoxon in scallion. Sens Actuators B Chem. 2023;398: 134584.

[135] Zhu HJ, Liu BX, Wang MZ, Pan JM, Xu LZ, Hu PW, Niu XH. Amorphous Fe-containing phosphotungstates featuring efficient peroxidase-like activity at neutral pH: toward portable swabs for pesticide detection with tandem catalytic amplification. Anal Chem. 2023;95:4776–85. 36862973 10.1021/acs.analchem.3c00008

[136] Wang YM, Li M, Wang ZR, Xu J, Zhao JJ, Gao ZD, Song YY. Photothermal effect-enhanced peroxidase-like performance for sensitive detection of organophosphorus pesticides on a visual test strip. Chem Eng J. 2023;476: 146329.

[137] Yi YH, Zhou X, Liao DY, Hou JL, Liu HD, Zhu GB. High peroxidase-mimicking metal-organic frameworks decorated with platinum nanozymes for the colorimetric detection of acetylcholine chloride and organophosphorus pesticides via enzyme cascade reaction. Inorg Chem. 2023;62:13929–36. 37583283 10.1021/acs.inorgchem.3c01844

[138] Wang JN, Wang XY, Wang M, Bian QH, Zhong JC. Novel Ce-based coordination polymer nanoparticles with excellent oxidase mimic activity applied for colorimetric assay to organophosphorus pesticides. Food Chem. 2022;397: 133810. 35917788 10.1016/j.foodchem.2022.133810

[139] Yang CL, Yu LH, Pang YH, Shen XF. A colorimetric sensing platform with smartphone for organophosphorus pesticides detection based on PANI-MnO_2_ nanozyme. Anal Chim Acta. 2023;1286: 342045. 38049237 10.1016/j.aca.2023.342045

[140] Liu FN, Li Z, Wei HY, Xu P, Kang G, Zhu SC, et al. Coordinatively unsaturated cobalt single-atom nanozymes for visual pesticides detection by smartphone-based platform. Nano Res. 2023;17:2298–307.

[141] Ge J, Yang LK, Li ZH, Wan Y, Mao DS, Deng RJ, et al. A colorimetric smartphone-based platform for pesticides detection using Fe-N/C single-atom nanozyme as oxidase mimetics. J Hazard Mater. 2022;436: 129199. 35643002 10.1016/j.jhazmat.2022.129199

[142] Ouyang H, Tu XM, Fu ZF, Wang WW, Fu SF, Zhu CZ, et al. Colorimetric and chemiluminescent dual-readout immunochromatographic assay for detection of pesticide residues utilizing g-C_3_N_4_/BiFeO_3_ nanocomposites. Biosens Bioelectron. 2018;106:43–9. 29414087 10.1016/j.bios.2018.01.033 PMC8649671

[143] Luo S, Gao JQ, Yuan HW, Yang J, Fan YH, Wang L, et al. Mn single-atom nanozymes with superior loading capability and superb superoxide dismutase-like activity for bioassay. Anal Chem. 2023;95:9366–72. 37276189 10.1021/acs.analchem.3c01623

[144] Zhang Y, Luo D, Zhou SK, Yang L, Yao WF, Cheng FF, et al. Analytical and biomedical applications of nanomaterials in Chinese herbal medicines research. Trends Anal Chem. 2022;156: 116690.

[145] Geng LG, Sun XD, Wang LD, Liu FP, Hu SQ, Zhao SL, Ye FG. Analyte-induced laccase-mimicking activity inhibition and conductivity enhancement of electroactive nanozymes for ratiometric electrochemical detection of thiram. J Hazard Mater. 2023;463: 132936. 37948782 10.1016/j.jhazmat.2023.132936

[146] Zhang CY, Peng LJ, Chen GY, Zhang H, Yang FQ. Investigation on the peroxidase-like activity of vitamin B6 and its applications in colorimetric detection of hydrogen peroxide and total antioxidant capacity evaluation. Molecules. 2022;27:4262. 35807507 10.3390/molecules27134262 PMC9268325

[147] Wang SN, Liu PC, Qin YM, Chen ZJ, Shen JC. Rapid synthesis of protein conjugated gold nanoclusters and their application in tea polyphenol sensing. Sens Actuators B Chem. 2015;223:178–85.

[148] Xu YX, Li PP, Hu XJ, Chen HY, Tang Y, Zhu Y, et al. Polyoxometalate nanostructures decorated with CuO nanoparticles for sensing ascorbic acid and Fe^2+^ ions. ACS Appl Nano Mater. 2021;4:8302–13.

[149] Sun S, Chen CY, Fu XY, Zhang YD, Wu XY, Hao JK, et al. Poly-β-cyclodextrin strengthen Pr_6_O_11_ porous oxidase mimic for dual-channel visual recognition of bioactive cysteine and Fe^2+^. Anal Bioanal Chem. 2024;416:1951–9. 38324071 10.1007/s00216-024-05192-1

[150] Cai XF, Liang MJ, Ma F, Mohamed SR, Goda AA, Dawood DH, et al. A direct competitive nanozyme-linked immunosorbent assay based on MnO_2_ nanosheets as a catalytic label for the determination of fumonisin B1. Anal Methods. 2021;13:5542–8. 34792520 10.1039/d1ay01654g

[151] Fan YX, Li D, Xie XY, Zhang Y, Jiang L, Huang B, Yang XP. Flower-like L-Cys-FeNiNPs nanozyme aptasensor for sensitive colorimetric detection of aflatoxin B1. Microchem J. 2024;197: 109842.

[152] Zhang XB, Wang FY, Li ZR, Hu B, Zheng QY, Piao YZ, et al. Dual-mode electrochemical/colorimetric microfluidic sensor integrated tetrahedral DNA nanostructures with Au/Ni-Co LDH NCs nanozyme for ultrasensitive detection of aflatoxin B1. Sens Actuators B Chem. 2023;393: 134322.

[153] Wu L, Zhou M, Wang YS, Liu JM. Nanozyme and aptamer-based immunosorbent assay for aflatoxin B1. J Hazard Mater. 2020;399: 123154. 32937727 10.1016/j.jhazmat.2020.123154

[154] He ZY, Zhang JX, Liu M, Meng YH. Polyvalent aptamer scaffold coordinating light-responsive oxidase-like nanozyme for sensitive detection of zearalenone. Food Chem. 2024;431: 136908. 37573743 10.1016/j.foodchem.2023.136908

[155] Zhu J, Xu WX, Yang Y, Kong RM, Wang JM. ssDNA-C_3_N_4_ conjugates-based nanozyme sensor array for discriminating mycotoxins. Microchim Acta. 2022;190:6. 10.1007/s00604-022-05593-y 36471087

[156] Xu X, Ma MY, Gao JX, Sun TX, Guo YH, Feng DM, Zhang L. Multifunctional Ni-NPC single-atom nanozyme for removal and smartphone-assisted visualization monitoring of carbamate pesticides. Inorg Chem. 2024;63:1225–35. 38163760 10.1021/acs.inorgchem.3c03642

[157] Wang Y, Yin L, Qu GX, Leung CH, Han L, Lu LH. Highly active single-atom nanozymes with high-loading iridium for sensitive detection of pesticides. Anal Chem. 2023;95:11960–8. 37530640 10.1021/acs.analchem.3c01569

